# Liquid Structure of a Water-in-Salt Electrolyte with a Remarkably Asymmetric Anion

**DOI:** 10.1021/acs.jpcb.1c06759

**Published:** 2021-11-05

**Authors:** Alessandro Triolo, Valerio Di Lisio, Fabrizio Lo Celso, Giovanni B. Appetecchi, Barbara Fazio, Philip Chater, Andrea Martinelli, Fabio Sciubba, Olga Russina

**Affiliations:** † Laboratorio Liquidi Ionici, Istituto Struttura della Materia, Consiglio Nazionale delle Ricerche (ISM-CNR), Rome 00133, Italy; ‡ Department of Chemistry, University of Rome Sapienza, Rome 00185, Italy; § Department of Physics and Chemistry, Università di Palermo, Palermo 90133, Italy; ∥ ENEA, SSPT-PROMAS-MATPRO Technical Unit, Rome 00123, Italy; ⊥ Istituto Processi Chimico-Fisici, Consiglio Nazionale delle Ricerche (IPCF-CNR), Messina 98158, Italy; # Diamond House, Harwell Science & Innovation Campus, Diamond Light Source, Ltd., Didcot OX11 0DE, U.K.; ∇ NMR-Based Metabolomics Laboratory (NMLab), Sapienza University of Rome, Rome 00185, Italy

## Abstract

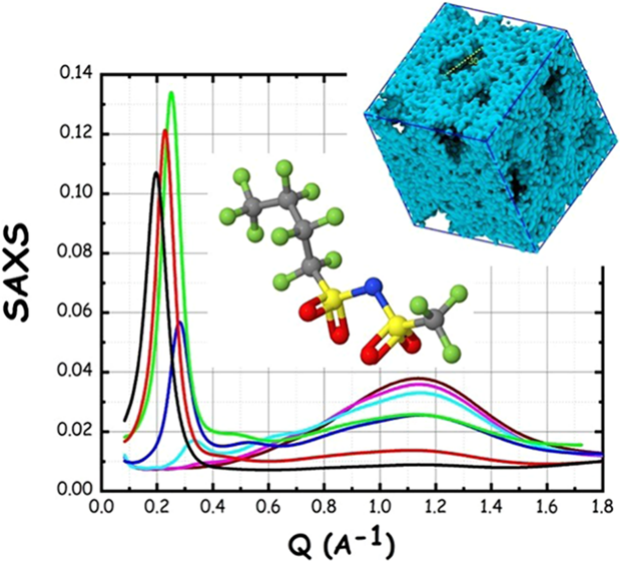

Water-in-salt systems, i.e., super-concentrated aqueous electrolytes, such as lithium bis(trifluoromethanesulfonyl)imide (21 mol/kg_water_), have been recently discovered to exhibit unexpectedly large electrochemical windows and high lithium transference numbers, thus paving the way to safe and sustainable charge storage devices. The peculiar transport features in these electrolytes are influenced by their intrinsically nanoseparated morphology, stemming from the anion hydrophobic nature and manifesting as nanosegregation between anions and water domains. The underlying mechanism behind this structure–dynamics correlation is, however, still a matter of strong debate. Here, we enhance the apolar nature of the anions, exploring the properties of the aqueous electrolytes of lithium salts with a strongly asymmetric anion, namely, (trifluoromethylsulfonyl)(nonafluorobutylsulfonyl) imide. Using a synergy of experimental and computational tools, we detect a remarkable level of structural heterogeneity at a mesoscopic level between anion-rich and water-rich domains. Such a ubiquitous sponge-like, bicontinuous morphology develops across the whole concentration range, evolving from large fluorinated globules at high dilution to a percolating fluorous matrix intercalated by water nanowires at super-concentrated regimes. Even at extremely concentrated conditions, a large population of fully hydrated lithium ions, with no anion coordination, is detected. One can then derive that the concomitant coexistence of (i) a mesoscopically segregated structure and (ii) fully hydrated lithium clusters disentangled from anion coordination enables the peculiar lithium diffusion features that characterize water-in-salt systems.

## Introduction

Super-concentrated aqueous electrolytes are presently the focus of intense research since the first experimental data appeared to reveal the unexpected performances of water-depleted salt solutions in the field of energy storage.^1^ Such systems are nowadays conventionally indicated as water-in-salt (WiS) mixtures to highlight the specific component ratio that characterizes them; in particular, one typically identifies aqueous electrolytes in such a way, when the salt to water ratio is larger than one, both by weight and volume. The interest in these systems stems from the enhanced and rather unexpected electrochemical stability of water-containing electrolytes in the specific concentration regime where WiS are defined. The narrow electrochemical stability of water (1.23 V) has traditionally limited aqueous electrolytes from application in energy storage devices. On the other hand, the use of more electrochemically stable organic media to support charge conduction in batteries is prone to potentially severe side effects related to solvent flammability and chemical stability. Accordingly, the discovery by Suo and co-workers that super-concentrated aqueous lithium bis(trifluoromethanesulfonyl)imide (LiTFSI) mixtures would perform an electrochemical stability up to ca. 3 V paved the way to a series of investigations aiming to rationalize and exploit this novel observation.^2−16^ Nowadays, however, several issues remain unexplored with respect to the morphology and the conduction mechanisms taking place in these unconventional media. Aqueous electrolytes have been studied in the past in dilute conditions, focusing on solvent-separated ion pairs, where water efficiently fully solvates the ionic species. Upon increasing the salt content, conductivity reaches a maximum and, due to increased viscosity, progressively decreases when the salt content reaches concentrations of the order of a few molal (mol_salt_/kg_solvent_). Accordingly, the highly concentrated regime that characterizes WiS systems has barely been explored in the past and only recently, more systematic studies are being developed in this new regime.^17−36^ Recent reviews have addressed the nature of the structural, dynamic, and electrochemical properties of these systems.^2,3,8,15,37−42^ Much of the structural investigations have been focused on the first WiS system, namely, LiTFSI-H_2_O, mostly due to LiTFSI high solubility in water (>20 m at 25 °C) and stability against hydrolysis.^1^ The phase diagram of this binary system has been characterized,^18^ showing the existence of a eutectic LiTFSI/H_2_O = 1:1, with a melting point at ca. −40 °C. This behavior has been recently framed in a more general trend involving other unconventional deep eutectic solvents formed by aqueous salt hydrates.^43^ At ca. 21 m (salt molar fraction = 0.275), the mixture has a melting point of 25 °C, representing the system with the highest Li content, which remains liquid at ambient conditions. LiTFSI was proposed as an electrolyte for aqueous lithium-ion batteries by Lux et al.,^44^ and its high concentration mixtures (*c* > 15 m) show interesting conductivity performances (5–10 mS/cm)^18^ and an appreciable electrochemical stability, at least up to 2 V.^1^ Nowadays, different options alternative to the LiTFSI-H_2_O WiS are being considered to enhance the resulting performance, including exploring Na- and K-based WiS^2,5,7,8,14,20,45−47^ or exploiting asymmetric anions and anion mixtures (leading to the so-called water in bisalt systems).^6,9,12,16,24,45,48,49^

**Scheme 1:**
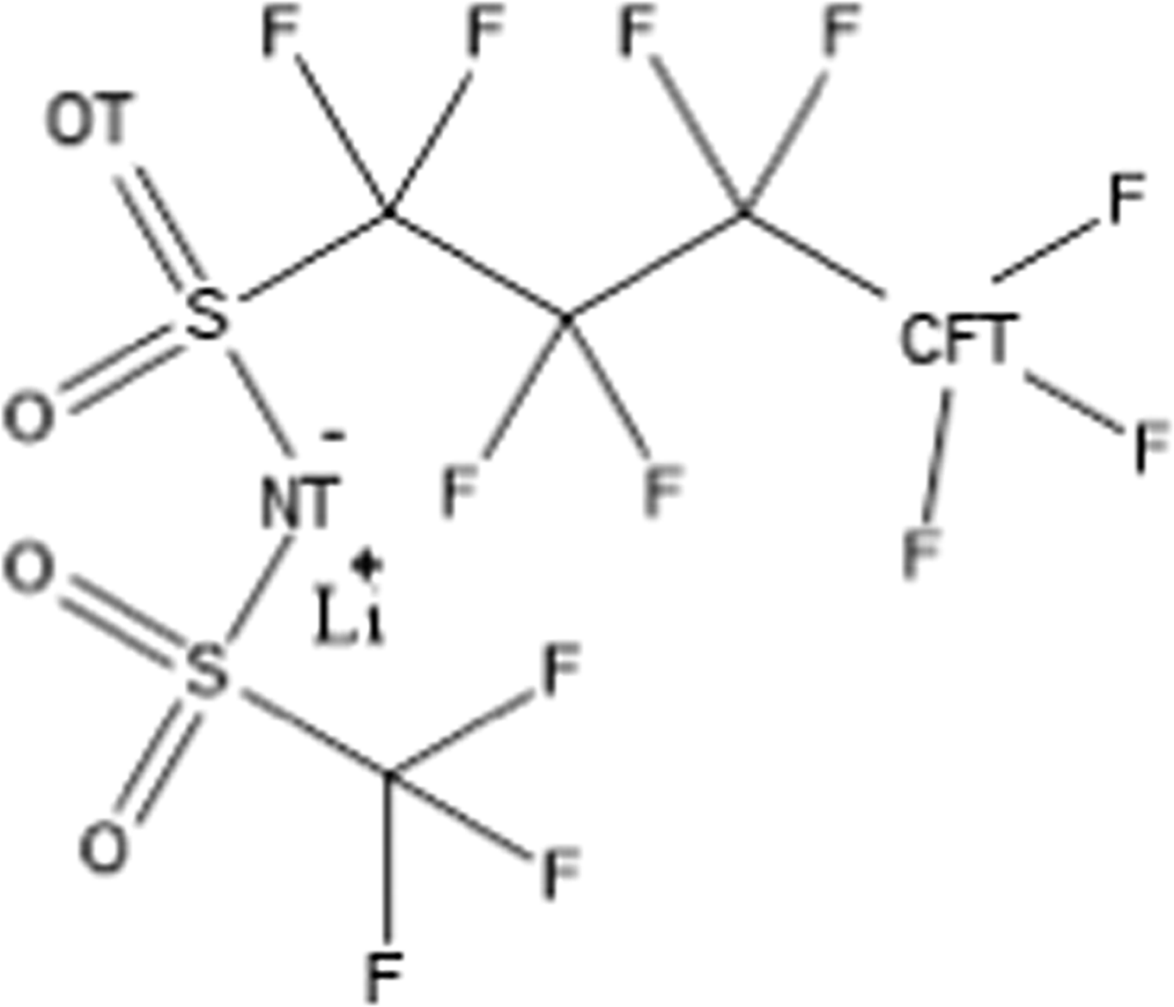
Chemical Structure of Lithium (Nonafluorobutanesulfonyl(trifluoromethanesulfonyl)imide (LiIM14) In the discussion of molecular dynamics simulation results, the anion’s oxygen, nitrogen, and terminal butyl carbon atoms are identified as OT, NT, and CFT, respectively.

Here, we explore an aqueous electrolyte system with a salt that is characterized by a remarkably asymmetric anion, i.e., a lithium salt with the anion being a member of the family of di(perfluoroalkyl-sulfonyl)imide, namely (trifluoromethylsulfonyl) (nonafluorobutylsulfonyl)imide (hereinafter indicated as [IM14]) (see Scheme 1). The high asymmetry of this anion makes it an ideal species to pair with cations that are prone to crystallization when paired with more conventional anions. In fact, we recently explored a range of ionic liquid compounds based on imidazolium or other cations paired with the [IM14] anion, highlighting their high tendency to remain in the liquid state even at very low temperatures.^50−54^ Very recently, a manuscript reported the role of anion size in the nanostructure of WiS systems, comparing the morphology detected in LiTFSI-based WiS with that in Li trifluoro-methanesulfonate (TfO) ones, highlighting the importance of the salt volume fraction in influencing the morphology.^48^ In this respect, the present choice for the anion represents an upper limit to the WiS systems studied so far.

In this contribution, we will show that LiIM14-based electrolytes are characterized by interesting properties from the point of view of the phase diagram (and hence the liquid state window) and electrochemistry (leading to super-concentrated electrolytes with appealing conductivity and electrochemical stability performances). We will further probe the structural organization in these electrolytes by exploiting the synergy between X-ray scattering, Raman and IR spectroscopies, calorimetry, electrochemical characterization, and molecular dynamics (MD) simulations to provide a robust characterization of the microscopic and mesoscopic organization in these systems. Due to the long perfluoro chain of the anion, a complex mesoscopic morphology develops, as detected by X-ray scattering techniques. The atomistic level description will be obtained by comparison between structural and spectroscopic information with MD results, providing a clear description of the role played by anion hydrophobicity in determining a nanoseparated morphology and of the enduring presence of fully hydrated lithium ions with no anions coordinating them, even at the most concentrated conditions. Despite the importance of chaotropic anions such as IM14 in enabling efficient WiS systems to be developed,^40^ so far, very little structural information exists on the organization of WiS based on salts different from LiTFSI.^28,42,55^ This work aims at expanding the spectrum of available salts that can be envisaged as electrochemically appealing WiS candidates, providing a novel insight into the structural role of long fluorous tails of the anions in affecting the ubiquitous structural heterogeneities in these systems.

## Experimental and Computational Details

### Chemicals

The lithium (trifluoromethylsulfonyl)(nonafluorobutylsulfonyl)imide, LiIM14, salt (see Scheme 1) was synthesized by reacting acidic (trifluoromethylsulfonyl)(nonafluorobutylsulfonyl)imide (HIM14, 3 M, 60 wt % solution in water) with lithium carbonate (Li_2_CO_3_, Fluka, >99.5 wt %) in slight excess (2 wt %) with respect to the stoichiometric amount for pushing the yield up to 100%, according to eq 1. Both the reagents were used as received.
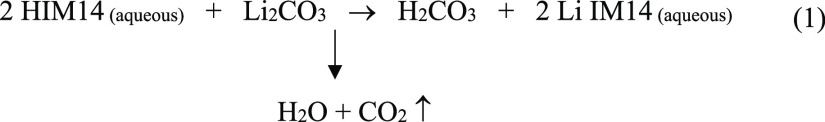


Lithium carbonate (solid) was slowly added, as the acid–base reaction is rather exothermal, to avoid excessive heat release. The so-obtained aqueous solution was stirred at room temperature for 30 min to promote CO_2_ removal, thus driving the reaction to completeness. Then, the water was removed in a rotary evaporator at 80 °C for 3–4 h, obtaining a solid, white LiIM14 salt. The Li_2_CO_3_ excess (within LiIM14) was removed by dissolving (stirring at room temperature) the salt in the minimal amount of absolute ethanol (VWR Chemicals, 100 wt %). Lithium carbonate, insoluble in ethanol, was separated by vacuum filtration (oil-free pump). Successively, the alcoholic LiIM14 solution was subjected to vacuum distillation (50 °C for 2 h) to remove ethanol. Finally, the LiIM14 salt was vacuum-dried at 120 °C overnight to reduce the water content below 5 ppm.

The LiIM14 solutions were prepared by dissolving the proper amount of salt in deionized (Millipore deionizer) water to obtain samples having a molality ranging from 1 to 20 m. The dissolution of LiIM14 in the most concentrated samples (i.e., 15 and 20 m) was promoted by stirring at 40–50 °C for 20–30 min. The mixtures were kept in sealed vials until ready for measurements.

### Differential Scanning Calorimetry (DSC)

DSC thermograms were acquired by a Mettler Toledo DSC 822e equipped with an FRS5 sensor and a liquid nitrogen cooler. The furnace was purged during the measurement with dry nitrogen at a flow rate of 30 mL/min. The samples of about 5 mg were weighed in a 40 μL aluminum pan and rapidly sealed. DSC scans comprised of cooling from 50 to −125 °C followed by heating from −125 up to 50 °C, with a heating/cooling rate of 2/10 °C/min.

### Density

Density data were obtained using a DM45 Mettler Toledo densimeter equipped with a vibrating tube with a resolution of 10^–5^ g/cc. Measurements were taken as a function of temperature that was controlled to be within 10^–3^ °C by means of a Peltier module. The instrument was calibrated with dry air and degassed-distilled water before performing the experiments.

### Wide-Angle X-ray Scattering (WAXS)

The total high-resolution X-ray scattering data were collected on the I15-1 beamline at Diamond Light Source, U.K., using X-rays of a wavelength of 0.309574 Å and a Perkin Elmer XRD 4343 CT detector. Such a setup allowed covering a *Q* range between 0.25 and 20 Å^–1^. The total scattering data were integrated to 1D using DAWN^56^ and then normalized and corrected to extract *I*(*Q*). The X-ray structure factors, *S*(*Q*), are normalized for the single atomic scattering, according to
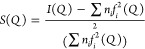
where *n_i_* and *f_i_*(*Q*) are the number concentration and the atomic scattering factors of the *i*th atomic species. The corresponding quantities are evaluated using molecular dynamics simulations for comparison purposes.

The samples were loaded into glue-sealed borosilicate capillaries of a 1.0 mm outer diameter; measurements were conducted at ambient conditions (ca. 20 °C). Additional data were collected at a Bruker D8 Advance diffractometer equipped with a Mo Kα X-ray tube (λ = 0.7107Å), using samples contained in 1.5 mm diameter quartz capillaries. In this case, the accessible angular range allowed covering between 0.6 and 15 Å^–1^.

### Small-Angle X-ray Scattering (SAXS)

Small-angle X-ray scattering (SAXS) measurements were performed at the SAXSLab Sapienza with a Xeuss 2.0 Q-Xoom system (Xenocs SA, Sassenage, France), equipped with a micro-focus Genix 3D X-ray source (λ = 0.1542 nm), a two-dimensional Pilatus3 R 300K detector, which can be placed at a variable distance from the sample. Calibration of the scattering vector *Q* range, where *Q* = (4π sin θ)/λ and 2θ is the scattering angle, was performed using a silver behenate standard.

Measurements with different sample–detector distances were performed so that the overall explored *Q* region was 0.1 < *Q* < 3 Å^–1^. The samples were loaded into a disposable quartz capillary with a nominal thickness of 1.0 mm and sealed with hot glue before placing them in the instrument sample chamber at reduced pressure (∼0.2 mbar). The beam size was defined through the two-pinhole collimation system equipped with scatterless slits to be 0.25 mm × 0.25 mm.

The two-dimensional scattering patterns were subtracted for the dark counting and then masked, azimuthally averaged, and normalized for transmitted beam intensity, exposure time, and subtended solid angle per pixel using FoxTrot software developed at SOLEIL. The one-dimensional *I*(*Q*) vs *Q* profiles were then subtracted for the capillary contribution.

The measurements were conducted at ambient temperature (ca. 20 °C), and the samples remained liquid and homogeneous during the whole length of the experiment.

### Electrochemical Properties

The ion transport properties of aqueous LiIM14 concentrated electrolytes were studied in terms of ionic conductivity vs temperature dependence. The measurements were performed at the temperature ranging from −40 to 80 °C at a very slow heating scan rate (1 °C/h) for better evidencing the phase transitions. A conductivity-meter AMEL 160, allowing to run impedance measurements at a fixed frequency (i.e., 1 Hz or 1 kHz, depending on the conduction value of the sample under test), was used, whereas the temperature control was performed using a climatic test chamber (Binder GmbH MK53). The electrolytes were housed in sealed glass conductivity cells (AMEL 192/K1) equipped with two porous platinum electrodes. The cell constant (depending on the geometric characteristics of the cell under test, ∼1.00 cm^–1^) was previously determined through a 0.1 N KCl aqueous solution having an exactly known conductivity value. Typical uncertainties on the conduction data are within 5%. The error bar in the conductivity plot (vide infra) falls within the data markers. To fully crystallize the aqueous LiIM14 electrolytes, the cells were dipped in liquid nitrogen for 60 s and then immediately transferred into the climatic chamber (previously set-up at −40 °C). This route was repeated until the frozen electrolytes remained solid at −40 °C. Finally, the cells were kept at −40 °C for at least 24 h prior to starting the conductivity measurements. The reproducibility of the conductivity data was verified by running the measurement set 2 times (from −40 to 80 °C) described above.

The anodic stability (toward oxidation) was evaluated by linear sweep voltammetry (LSV) carried out on a symmetrical, platinum, two-electrode (thickness and diameter equal to 100 μm and 10 mm, respectively) cells. The Pt electrodes, sandwiching a glass fiber separator (10 mm diameter), were housed within T-shape poly(propylene) containers, using steel rods (10 mm diameter) as the current collectors. The electrolytes under test (about 1 mL) were loaded into the cell containers, which were then locked to avoid liquid leakage. The measurements were carried out at 1 mV/s and room temperature, using a PAR 2273 galvanostat/potentiostat, by scanning the cell voltage from the OCV value toward more positive (anodic limit) voltages. Clean electrodes and fresh cells were used for each test. To confirm the reproducibility of the results, the LSV tests were run at least twice on different fresh cells.

### Raman and Infrared Spectroscopy

Raman spectra were acquired at room temperature using a LabRam HR800 Raman Spectrometer (Horiba Jobin Yvon), equipped with an Olympus BX41-microscope accessorized for macro investigation (a 4× magnification objective and a multipass cell holder). The He–Ne laser beam at λ = 632.8 nm was focused with a power of 3 mW on the sample placed in a glass cuvette. The Raman scattered light was collected in a backscattering configuration via the same illumination objective, dispersed by a 600 L/mm grating, and then detected through a Peltier-cooled silicon CCD (Synapse by Horiba Jobin Yvon). The spectra were typically acquired with integration times of 120 s.

Infrared spectra of LiIM14–water mixtures were acquired at room temperature and in the transmission mode using a Nicolet FTIR 6700 Spectrometer by Thermo Fisher Scientific. Solutions were pressed between two ZnSe windows (2 mm thickness) without using a spacer to avoid signal saturation and held in position by a liquid cell holder purchased from Specac. Spectra were recorded in the 4000–400 cm^–1^ range by co-adding 100 scans at a resolution of 2 cm^–1^.

#### MCR-ALS Spectral Decomposition

FTIR and Raman spectra in the water absorption region were analyzed using a Multivariate Curve Resolution-constrained Alternating Least Squares (MCR-ALS) bilinear prediction model^57,58^ (mcr_toolbox 2 add-on) implemented in MATLAB software. Using the MCR-ALS model, Infrared and Raman spectra of LiIM14 solutions were decomposed into a linear combination of several absorbing species contributing to the concentration-dependent spectral variation. Two data sets were created and analyzed separately, the first containing FTIR absorbance spectra in a spectral range composed of the 4000–2850 cm^–1^ OH stretching region and the 1850–1450 cm^–1^ HOH bending region. A second dataset was formed by the Raman intensities spectra in the OH stretching region (4000–2800 cm^–1^). The MCR-ALS algorithm decomposed an initial dataset in a product of two smaller matrices, the first containing the spectral profiles of the absorbing species and the second comprised of the concentration profiles (or spectral coefficients). First, an MCR-ALS model comprising three spectral components was chosen by performing a preliminary principal component analysis. This model accounts for 99.7 and 99.8% of the total spectral variance for the FTIR and Raman data sets, respectively. The initial estimation of the three spectral profiles was performed by singular value decomposition (SVD). Finally, the convergence of the iterative fitting procedure was achieved when the standard deviation fell below 0.0001 for both FTIR and Raman data sets. To obtain meaningful information, a non-negative constraint was applied to both concentration and spectral profiles, and closure constraint was applied to keep the sum of the concentration profiles equal to 1.^59^ Final fittings were characterized by a LOF% (lack of fit) parameter of 2.3% for FTIR and 2.7% for Raman data sets.

### Molecular Dynamics (MD) Simulations

Classical MD simulations for LiIM14 WiS were performed at different concentrations consistent with experimental data sets. In particular, we simulated systems with *c* = 1, 2, 5, 7, 10, 15, and 20 m for the LiIM14–H_2_O system.

MD simulations were performed using GROMACS 2018.3 package software.^60,61^ Bonded and nonbonded parameters for the IM14 anion were described using an all-atoms potential;^62−64^ the SPCE water model was used for the solvent.^65^ The Li-ion potential was taken from ref (66).

The simulations for LiIM14–water solutions were performed using cubic boxes; the initial edge size was fixed between 8.5 and 10 nm depending on the concentration; periodic boundary conditions were applied. We stress that large simulation boxes were required to satisfactorily reproduce the experimentally determined structural properties, and more conventional smaller boxes would have missed to grasp fundamental structural features. The initial configurations were created by Packmol software.^67^ The equilibration procedure was performed in several steps, starting from an NVT simulation at 400 K and scaled partial charges (10% of the original ones), followed by a series of NPT runs lowering the temperature progressively (from 400 to 350 K) and increasing the charges to their final value (80% of the original ones) at 298 K and 1 bar after a 6 ns run. After the equilibration phase, each system was run for at least 150 ns for the production run, and then a further trajectory of 4 ns was saved at a frequency of 2 ps for the calculation of structural properties. The production simulations were always checked vs the energy profile. During the production runs for the temperature coupling, we used a velocity rescaling thermostat^68^ (with a time coupling constant of 0.1 ps), while for the pressure coupling, we used a Parrinello–Rahman barostat^69^ (1 ps for the relaxation constant). The leap-frog algorithm with a 1 fs time step was used for integrating the equations of motion. Cut-offs for the Lennard-Jones and real space part of the Coulombic interactions were set to 15 Å. For the electrostatic interactions, the particle mesh Ewald (PME) summation method^70,71^ was used, with an interpolation order of 6 and 0.08 nm of FFT grid spacing. Selected graphs were done using VMD.^72^ Weighted and partial structure factors were computed using in-house developed software, while the selected pair correlation function and angular distribution function were obtained by TRAVIS.^73−75^ Analysis of the shortest contiguous hydrogen-bond path between every pair of water molecules as well as the path between hydrogen-bonded water molecules and water molecules connected via lithium interaction has been conducted using ChemNetworks software.^76^

## Results and Discussion

**Figure 1:**
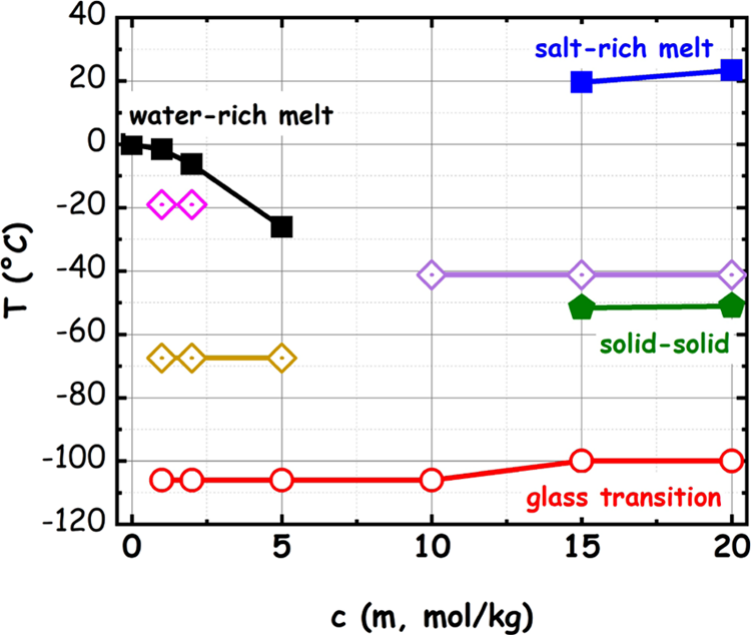
Phase diagram of the LiIM14–H_2_O system obtained from calorimetric measurements. Full symbols refer to strong endothermic transitions; open symbols refer to the liquid–glass transition; dotted symbols refer to weak, spike-like features. Lines are guides for the eye.

All of the solutions probed in the present study are thermodynamically stable in their liquid state above 25 °C. The LiIM14–water electrolytes are herein reported for the first time, and their phase diagram has not been published so far. Figure S-1 shows the DSC traces for LiIM14–H_2_O mixtures, in the concentration range between 1 and 20 m, at a heating rate of 2 °C/min. This information leads to a proposal for the phase diagram of the LiIM14–water system over the presently reported concentration range (see Figure 1). We mention herein that both WiS with LiIM14 at *c* = 15 and 20 m, when cooled from the melt, remained in the liquid state at temperatures around 20 °C, where some measurements were conducted, despite their melting point being slightly above this value: accordingly, we likely characterized a slightly supercooled state of these mixtures in our experimental X-ray studies. The LiTFSI–water phase diagram is highly related to the presently reported LiIM14–water one. Ding and Xu^18^ reported the whole phase diagram for aqueous LiTFSI for 0 ≤ *x*_LiTFSI_ ≤ 1, at ambient pressure. For this study, they used samples containing carbon microbeads to facilitate nucleation events that might lead to a safer characterization of solid phases. In our present study, we do not use such an option: accordingly, some crystallization events might have been overlooked. LiTFSI–H_2_O features an articulated phase diagram, and two different hydrates have been detected therein: namely, LiTFSI·(H_2_O)_4_ and LiTFSI·(H_2_O) that have been assumed to be formed by positively charged hydrated lithium ions paired with the anion. Our present study does not allow extracting this information due to the limited number of samples considered. We notice that upon increasing the salt content in neat water, a progressive decrease of the water-rich (*c* ≤ 5 m) mixture melting point is observed. These mixtures are characterized by two further solid–solid transitions at −19 and −67.5 °C that appear as very weak calorimetric features (presumably due to incomplete transformations) and, eventually, at low enough temperature, by a glass transition event at −106 °C (without appreciable concentration dependence). At more concentrated conditions (*c* = 10 m), only a very tiny feature is observed at −41.2 °C, and otherwise, the sample is subjected to a liquid–glass transition at −106 °C. This specific concentration looks peculiar, as no strong endothermic events seem to occur. It corresponds to an *x*_LiIM14_ = 0.2 salt molar fraction and seems relatively easy to be supercooled to the amorphous state, without intervening crystallization, seemingly leading to a eutectic composition (this might provide a hint to the existence of stable hydrates with stoichiometry: LiIM14·(H_2_O)_4_; further research is active on this topic). A higher salt content leads to a shift of glass transition toward higher temperature (i.e., a more rigid environment), and a cold crystallization and subsequent melting can be observed at higher temperatures. Eventually, a solid–liquid transition occurs at approximately room temperature. These systems will need to be further investigated with greater detail; nevertheless, valuable information on the liquid state conditions and on the existence of several crystalline phases can be safely assessed.

**Figure 2:**
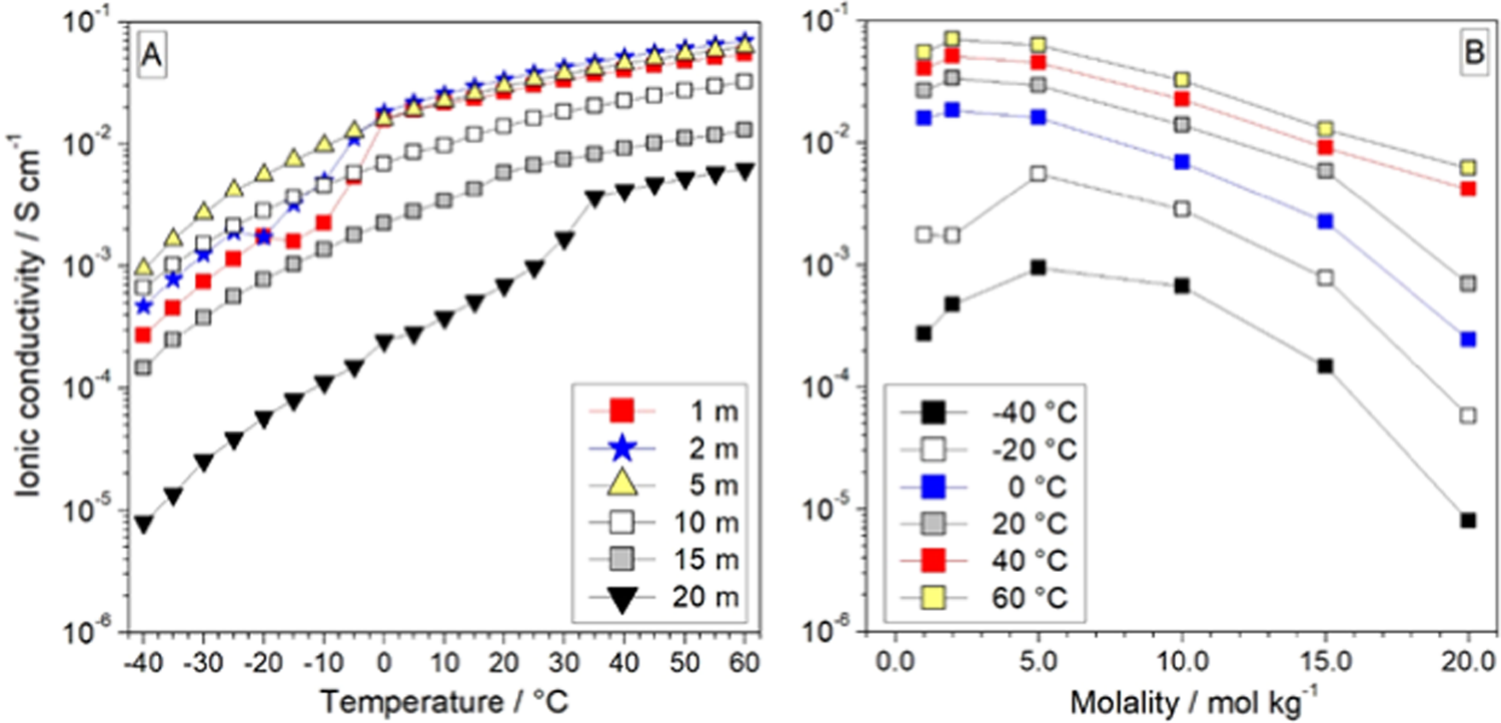
(a) Ionic conductivity of the LiIM14–H_2_O system as a function of temperature for different salt contents; (b) different isotherms for the ionic conductivity of the LiIM14–H_2_O system, as a function of salt content.

The temperature dependence of the ionic conductivity for selected LiIM14–water electrolytes is reported in Figure 2a. Analogous behavior and high reproducible conduction values were obtained from two measurement sets, indicating good reliability of the results. All investigated electrolyte samples, with the exception of the 20 m one, show conductivity values ranging from 10^–4^ to 10^–3^ S/cm, i.e., of interest for practical applications, already at −40 °C. This experimental evidence, supporting a gained ion mobility in the frozen state likely due to the very large steric hindrance of the IM14 anion, makes the LiIM14 WiS solutions appealing for electrochemical devices operating at very low temperatures. The conductivity behavior of the *c* = 1 and 2 m samples in the range between −25 and −15 °C might confirm the existence of solid–solid phase transitions and/or different ion rearrangement prior to the melting temperature in this concentration range at ca. −19 °C (Figure S-1), in which the ions show lower mobility.

Conversely, no evident conductivity jump is detected for the *c* = 5 and 10 m samples within the whole investigated temperature range. The more concentrated electrolytes (*c* = 15 and 20 m) exhibit a conductivity increase from −40 °C up to room temperature, likely ascribable to the progressive structural reorganization of ions and/or solid–solid phase transitions. Around 20 and 30 °C increase in moderate conductivity (particularly for the *c* = 15 m sample) is observed, indicating melting of the *c* = 15 and 20 m electrolytes, consistently with calorimetric results. In the molten state, the ionic conductivity of the LiIM14–H_2_O solutions exhibits a Vogel–Fulcher–Tammann trend, which displays, as expected, a progressive increase with the temperature.^77−79^

Figure 2b plots the dependence, at different temperatures, of the ionic conductivity from the solution molality. A bell behavior is observed with a maximum value located between *c* = 2 and 5 m, similar to the behavior observed for the LiTFSI–H_2_O system.^1,80^ The conductivity (σ) of electrolytes such as the LiIM14-H_2_O solutions is governed by the following equation
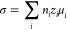
where *n*_*i*_ represents the charge carrier number, *z*_*i*_ is the ionic charge, and μ is the mobility of the *i*th ion species. At low LiIM14 concentrations (*c* < 2 m), the electrolyte conductivity is found to increase with the lithium salt molality due to the increase of the charge carrier number. Also, the increase of the LiIM14 molality leads to ion mobility decrease, but this effect is fully counterbalanced by the increase of the charge carrier number, overall enhancing the conductivity value. Conversely, at higher concentrations (*c* > 5 m), the increase of the lithium salt molality leads to the formation of multiple ions and/or neutral ionic couples, this progressive lowering of the overall free charge carrier number and, therefore, the conduction value of the water solution, thus leading to a maximum in conductivity as the salt content increases. At low temperatures (*T* ≤ 0 °C), the aqueous LiIM14 electrolytes show a maximum conductivity around a salt molality of 5 m (at this condition, some of the samples are still in the solid state), whereas above 30 °C (i.e., when all solutions are in the molten state), such a maximum value is seen shifting to *c* = 2 m. It is to be noted that all investigated LiIM14–H_2_O solutions exhibit ion conduction values of interest for practical electrochemical devices (>10^–3^ S/cm) at −20 °C, making these electrolyte systems appealing for low-temperature applications.

**Figure 3:**
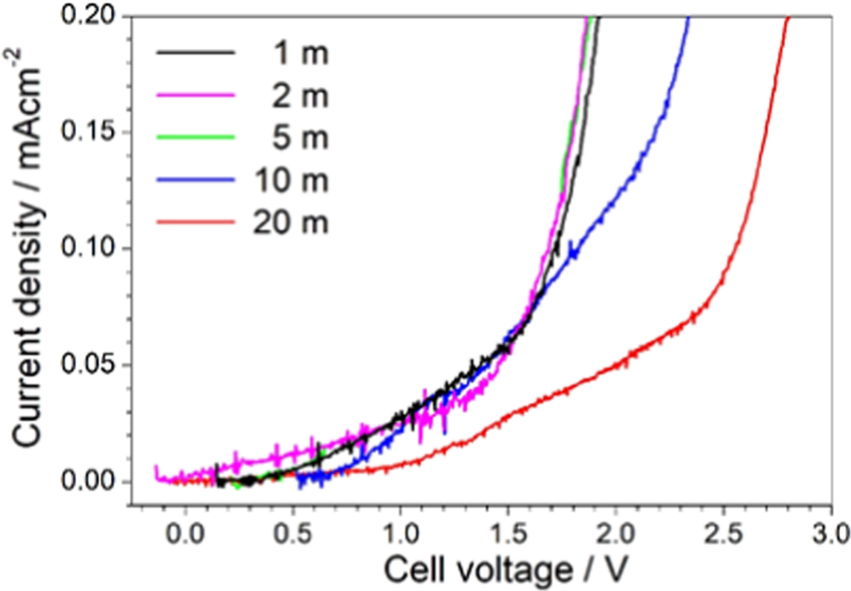
Anodic linear sweep voltammetry traces obtained for the LiIM14–H_2_O system at room temperature for different salt contents.

Together with ion transport properties, electrochemical stability is another important electrolyte property in view of its application in practical devices. Figure 3 displays the anodic linear sweep voltammetry (LSV) traces obtained for the investigated LiIM14–H_2_O systems. A sudden current increase, observed in the voltage range from 1.5 to 2.5 V, indicates massive degradation (oxidation) of the electrolyte samples. Similar electrochemical behavior (i.e., no practical improvement in terms of anodic stability) is detected up to *c* = 5 m, whereas a progressive shift of the anodic limit voltage is observed with the increase of the LiIM14 concentration above *c* = 5 m. As known, the Li^+^ cations, due to their high surface charge density (ascribable to their small steric hindrance), can strongly coordinate the polar water molecules. Thus, the increase of the LiIM14 concentration leads to a progressive decrease of the fraction of free (i.e., nonbounded to the lithium salt) water molecules (vide infra). Up to *c* = 5 m, the fraction of free water within the electrolyte sample is remarkable and it starts to degrade around 1.0 V according to the reaction (i.e., redox potential equal to −0.828 V vs 2H^+^/H_2_)
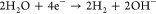


Above *c* = 5 m, the free-water fraction is progressively decreasing, and especially at very high salt molality values (*c* = 20 m), all water molecules are practically involved in the lithium salt solvation (vide infra). Therefore, the voltammetry results seem to provide support for the existence of a lithium salt concentration threshold, which governs the electrochemical behavior of these highly concentrated aqueous electrolyte systems. Below this threshold, the free-water content is relevant and drives the anodic stability of the solutions: no practical gain in terms of anodic limit voltage is observed. Above this salt concentration, the free-solvent fraction is negligible, i.e., almost the overall aqueous solvent amount is involved in strong coordination of the LiIM_14_ salt (especially of the Li^+^ cations), and the anodic stability is found to increase with increasing the lithium salt molality. Spectroscopic and computational evidence in the next sections will confirm this behavior. Therefore, very large LiIM14 concentrations, fully involving the whole aqueous solvent in the solvation of ions, are able to shield the H_2_O molecules from oxidation processes, thus enhancing the anodic stability of the aqueous electrolytes. Similar behavior was previously observed for concentrated aqueous solutions based on LiTFSI.^1^ Therefore, even if the electrochemical stability is not still sufficiently wide for applications in lithium battery systems^81^ operating at high voltages (i.e., above 4 V), as also reported in the literature,^1^ the very high molar concentration of the LiIM14 salt is able to enhance the robustness of the aqueous solution toward oxidation. Although other effects play a role in the electrochemical stability of super-concentrated WiS (see, e.g., ref (10)), in any case, this feature makes the present WiS system a promising class of materials for future applications in electrochemical energy storage systems.^2,3,8,11,12^

**Figure 4:**
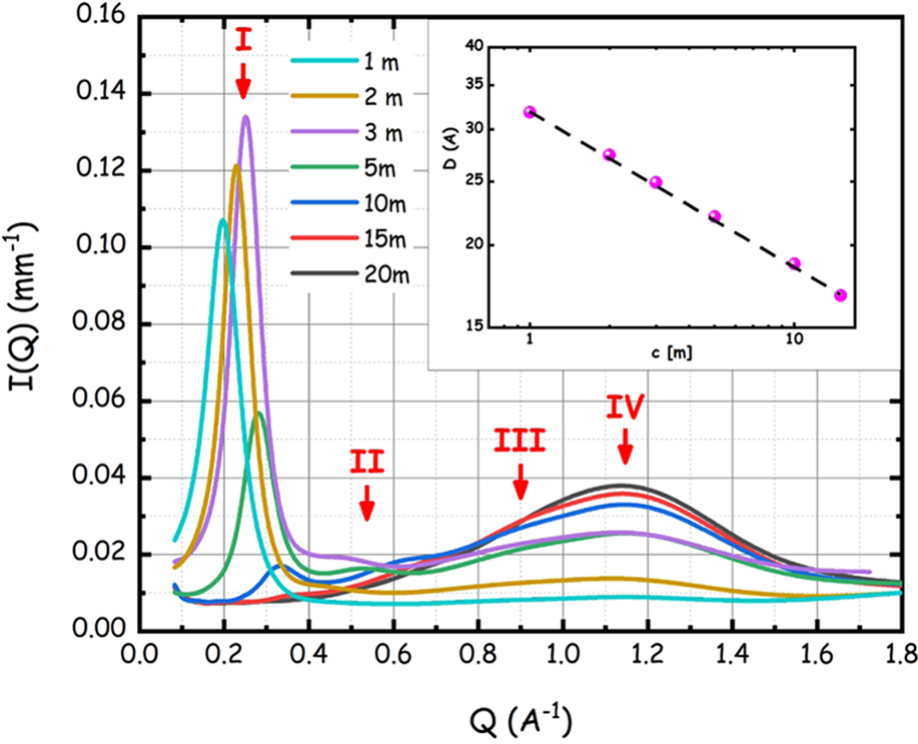
Small-angle X-ray scattering patterns for the LiIM14–H_2_O system at room conditions as a function of salt content. The roman numbers refer to the four different peaks observed in the patterns. In the inset, the log–log salt concentration dependence for the characteristic size associated with peak I is reported.

The selected electrolyte system has been characterized in terms of its density properties as a function of temperature between 20 and 60 °C. These data are reported in Figure S-2. In the case of *c* = 20 m, the sample at 20 °C is in a supercooled condition that could be reliably characterized, without intervening crystallization, during the measurements. We next probed morphological properties. Figure 4 shows the small-angle X-ray scattering (SAXS) data sets collected for the series of LiIM14–water in the concentration range between *c* = 1 and 20 m at ca. 20 °C. The LiIM14–water SAXS patterns are characterized by four peaks in the probed *Q* range (a strong peak (I) at *Q* values < 0.4 Å^–1^, one in the range of 0.4–0.7 Å^–1^ (II) and two peaks (III and IV) at *Q* values above 0.7 Å^–1^, as outlined in the figure by the red arrows). It emerges clearly that at a high/medium water content (*c* ≤ 10 m), a very strong scattering halo (peak I) develops at low *Q* values (*Q* < 0.4 Å^–1^). The intermediate peak (peak II) is appreciable, although with weaker amplitude, across the whole concentration range. Such a situation is different from the ones reported in the recent past for the case of the LiTFSI–water system. Borodin et al. observed a peak (likely corresponding to our present peak I) in LiTFSI WiS (with *c* = 21 m, with D_2_O), using small-angle neutron scattering data.^13^ Recently, a report from Zhang et al. showed high-energy X-ray scattering data from a series of LiTFSI–water systems in the concentration range between 1 and 20 m.^28^ Their data allow detecting the presence of two peaks in the range between 0.2 and 2 Å^–1^. These data are similar to the ones reported by Liu et al. for the same system.^55^ The neutron weighted simulated patterns reported by Borodin et al. for LiTFSI WiS, with the concentration ranging from 5 up to 21 m, indicate a progressive growth in the amplitude of the low *Q* feature but their simulations do not seem to indicate an appreciable change in the peak position, between *c* = 5 and 21 m.^13^ The data presented by Zhang et al. do not allow detecting this behavior, as they are vertically shifted; they observe, however, a distinct shift in the peak position.^28^ Very recently, Tan et al. reported neutron and X-ray scattering data from the LiTFSI–water system at *c* = 0.3 and 21 m.^22^ Their study highlighted the presence of peak I (centered at 0.4 Å^–1^) in concentrated solution using both X-ray and neutron scattering, but they claim that peak I is not present in dilute solutions. Horwitz et al. monitored peak I evolution between *c* = 4 and 21 m for LiTFSI–water by neutron scattering using D_2_O.^48^ Our present results on LiIM14–water electrolytes indicate the progressive development of the distinct low *Q* X-ray scattering peak I upon increasing the water content, whose position clearly shifts with the concentration. In Figure S-3, we show the data of Figure 4 in log–log scale and highlight the concentration dependence of peaks positions. It emerges that peaks III and IV positions show only a minor concentration dependence. On the other hand, both peaks I and II appreciably shift toward higher *Q* values upon increasing the salt content. This behavior is similar to what Zhang et al. reported for their peaks B and A, respectively, in their paper.^28^ The behavior is also similar to the one highlighted by Horwitz et al. in their recent paper.^48^

Peak positions for peak I, *Q*_p_, have been determined as a function of salt concentration by fitting the experimental data with a Gaussian function and the corresponding real space sizes estimated as *D* = 2π/*Q*_p_ are reported in the inset of Figure 4. The linear trend of log *D* vs log *c* in the concentration range (1 ≤ *c* ([m]) ≤ 15) probed by the present study can be noticed. The concentration dependence of peak I amplitude is noteworthy; while our data show that the peak occurs ubiquitously in the probed concentration window, its amplitude shows a maximum at ca. *c* = 3 m. Accordingly, at odds with the observation done by Tan et al.,^22^ the structural heterogeneities leading to the appearance of peak I are present over the whole probed concentration range. In this context, we also mention the recent report from Liu et al., where a large set of concentrations of LiTFSI–water mixtures has been studied by small-angle X-ray scattering (SAXS), confirming our present findings that peak I is stronger at more dilute conditions and, by increasing the salt content, its amplitude tends to decrease (and even vanish) and its position shifts to higher *Q* values.^55^

Overall, then, we can state that, apart from the peak amplitude, we do not observe a drastic differentiation between salt-in-water and water-in-salt regimes concerning the low *Q* peak in this class of material. Such an observation is important to properly address the attention in the exploration of lithium diffusivity in WiS. The present results indicate the existence of structural heterogeneities of the order of several nanometers, as revealed by X-ray scattering, whose size depends on the electrolyte composition. Such heterogeneities are present across the explored concentration window in the present electrolytes. This scenario will be confirmed by molecular dynamics simulations later on. At the present stage, we can, however, propose the existence of a sponge-like, bicontinuous morphology that characterizes the mutual distribution of self-excluding domains of apolar, fluorinated anions and water in these electrolyte systems.

The series of LiIM14–water samples have also been characterized by synchrotron high-energy X-ray diffraction, aiming at accessing a larger *Q* range than the one accessible via the SAXS technique. These data are of course compatible with the SAXS ones in their common *Q* range, but they also provide information on shorter-range structural correlations occurring in the liquid samples by accessing *Q* values as high as 20 Å^–1^. Typically, these data sets are used to provide experimental validation of the structural properties as extracted via molecular dynamics simulation, which can be judged by the quality of the agreement between experimentally and computationally derived static structure factors, *S*(*Q*).

**Figure 5:**
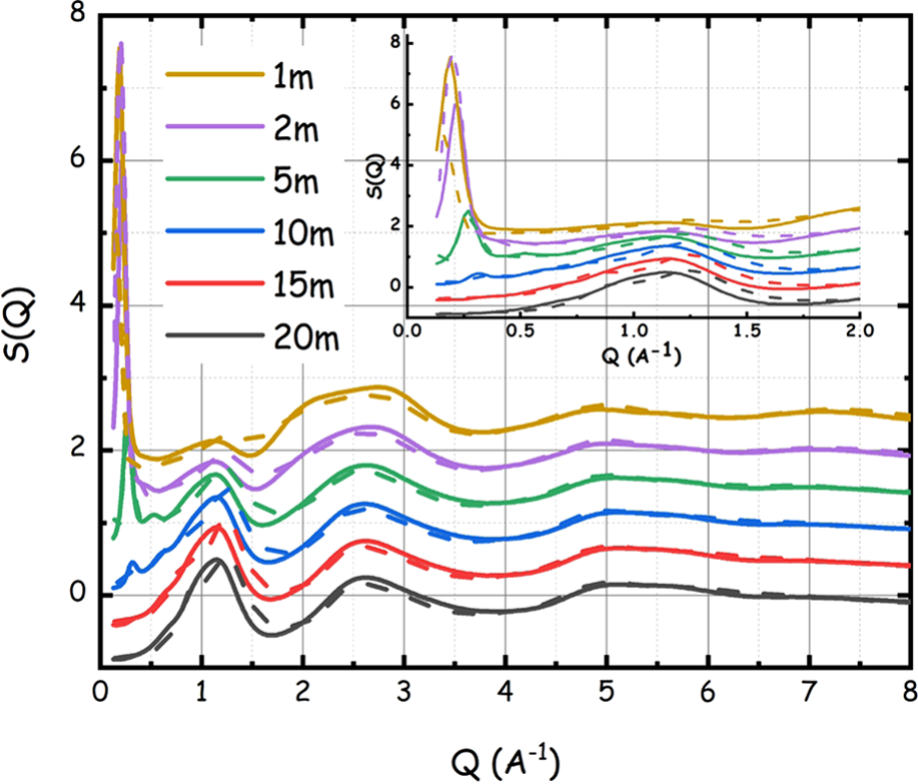
Experimental (continuous lines) and MD-derived (dashed lines) wide-angle X-ray scattering patterns from the LiIM14–H_2_O system for different salt contents at ambient conditions. In the inset, the low *Q* portion of the spectra is highlighted.

Figure 5 reports such a comparison between the measured *S*(*Q*) (over the range 0.1 ≤ *Q* (Å^–1^) ≤ 8) and the corresponding patterns as obtained from the MD simulations. The latter nicely account for all of the relevant experimental features and, especially, for the emerging of the strong peak at low *Q* values, upon diluting the mixtures. We stress that to satisfactorily reproduce the low *Q* scattering features, a large simulation box is required. Here, the use of box sizes of the order of 80–100 Å turned out to be fundamental for the purpose. More conventional sizes (e.g., 30–50 Å) would either miss to reproduce or wrongly estimate the position and amplitude of such features.

For the sake of completeness, in Figure S-4, we report our molecular dynamics computed *S*(*Q*) as would be obtained from neutron scattering experiments using either H_2_O or D_2_O. Therein, one can appreciate the ubiquitous presence of peak I over the whole probed concentration window, thus supporting the above discussion.

Together with the comparison between experimental and computed X-ray scattering patterns, we further validated the presently reported MD simulations with the experimental values of density at 25 °C. The agreement is very good and is reported in Figure S-5. This robust experimental validation of the simulations makes us confidant in their exploitation for extracting accurate structural information at the atomistic level.

**Figure 6:**
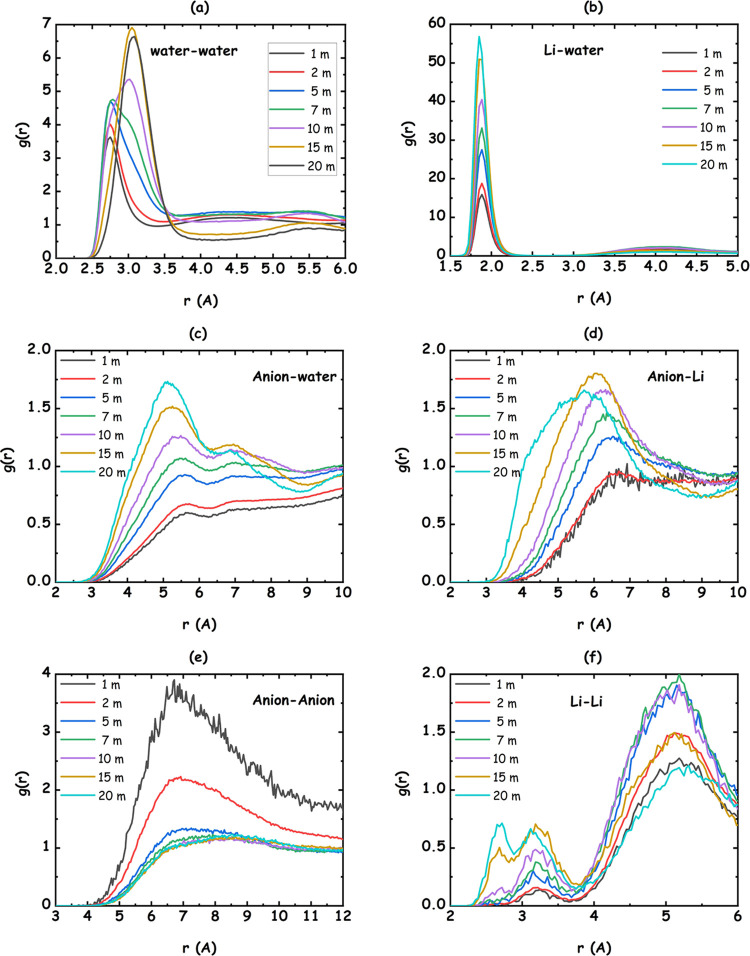
MD-derived center of mass pair distribution functions for the different species in the LiIM14–H_2_O systems, for different salt contents: (a) water–water; (b) lithium–water; (c) anion–water; (d) lithium–anion; (e) anion–anion, and (f) lithium–lithium correlations are shown.

**Figure 7:**
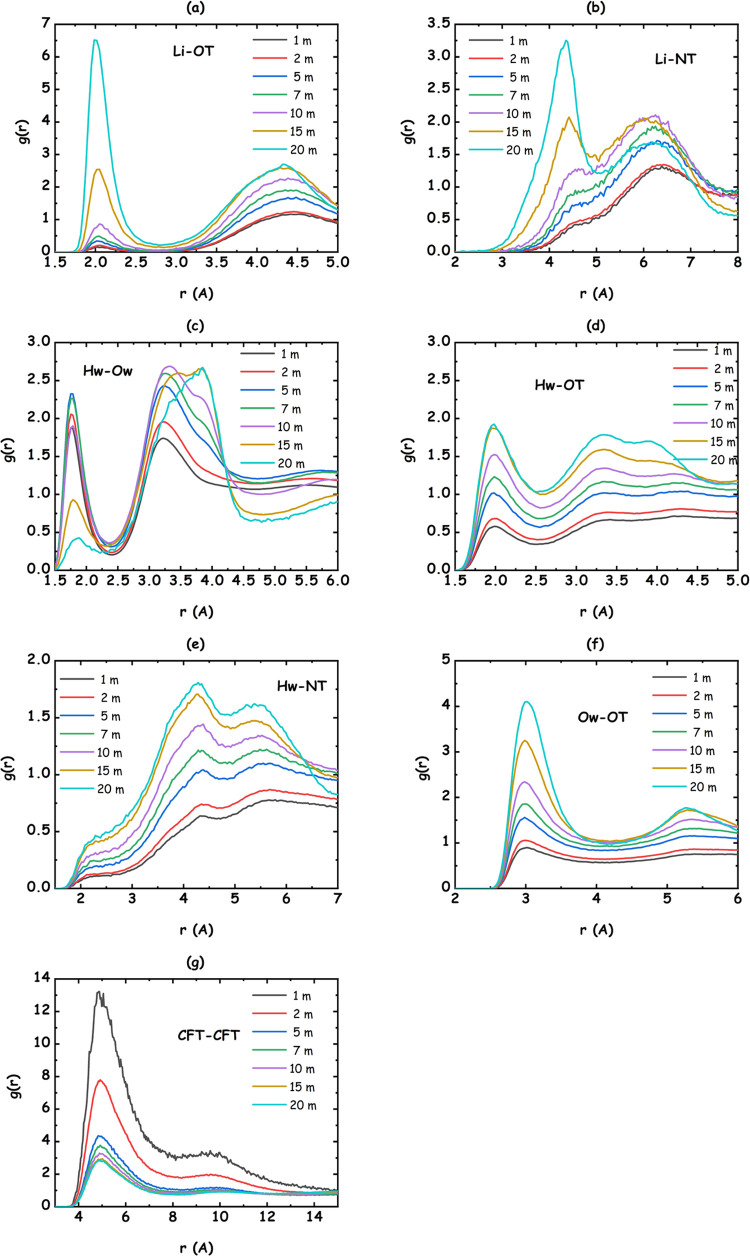
Selected, MD-derived pair distribution functions for the different species in the LiIM14–H_2_O systems, for different salt contents: (a) Li–OT; (b) Li–NT; (c) Ow–Hw; (d) OT–Hw; (e) NT–Hw; (f) OT–Ow, and (g) CFT–CFT correlations are shown. Ow and Hw refer to water’s oxygen and hydrogen atoms; OT, NT, and CFT refer to anion’s oxygen, nitrogen, and terminal butyl carbon atomic species (see Figure 1).

As preliminary information, we interrogated the MD simulations to extract the pair distribution functions (PDF) related to the three species centers of mass (CoM) mutual correlations for the different investigated WiS. These data are plotted in Figure 6a–f, where the concentration dependence of the self and cross-correlations are reported for the three species: namely, water, lithium, and the [IM14] anion. Other relevant PDFs related to interatomic correlations are presented in Figure 7 (corresponding figures containing the running coordination numbers are reported in Figures S-6 and S-7).

In general, the observed trends tend to be similar to recent results from two different groups, which focused on structural properties of LiTFSI–water system.

### Water–Water Correlations

Considering the case of water–water correlations (Figure 6a), while dilute mixtures are characterized by a simple peak centered at 2.75 Å, reflecting bulk water’s tetrahedral organization, on the other hand, upon increasing the salt concentration, a progressively bimodal distribution (that is clearly visible at the two concentrations *c* = 7 and 10 m) will eventually evolve into a single peak centered at 3.1 Å, reflecting a change in water–water correlations. Following the rationalization for this behavior provided by Zhang et al.,^28^ we observe that in pure water and in dilute LiIM14 mixtures, the conventional tetrahedral organization of water molecules surrounding a reference water molecule is reflected by the peaks at 2.75 and 4.5 Å. On the other hand, the progressively increasing lithium content will lead to a decrease of the bulk-water population and the development of lithium-mediated water–water correlations with a characteristic water–water distance of the order of 3.2 Å.^28^ It is important to note that lithium-mediated neighbor waters are not directly interacting through the hydrogen-bonding interaction between themselves. Accordingly, the shift and splitting of the water–water PDF peak reflects a progressive change in the nature of water environments in the solutions. In Figure S-8, we show the distribution numbers of water oxygens, Ow, coordinating a reference Ow, as a function of salt content. Figure S-8a shows the distribution of Ow coordination numbers obtained inside a shell of 3.3 Å (that is, the typical O···O distance between hydrogen-bonded Ow’s in bulk water). One can notice that upon increasing the salt content, the local water environment remains appreciably uninfluenced up to *c* = 2 m; above this value, one notices a progressive shift toward a smaller number of coordinating waters. Figure S-8b highlights that the average Ow coordination number around a reference Ow is found to progressively decrease down to ca. 2 when increasing the salt content. Moreover, one finds a dramatic increase of the number of reference Ow’s with no surrounding hydrogen-bonded water molecules, with 50% water molecules not bound to any other one via HB, already at *c* = 10 m (Figure S-8c). Such a situation is reflected by the concentration trend observed for the orientational tetrahedral order (OTO) parameter^82^ that is reported in Figure S-9. Therein one can observe that dilute solutions are characterized by a water network surrounding a reference water molecule resembling the tetrahedral order observed in neat water. However, when the salt content increases, the OTO parameter strongly deviates from the neat water behavior.

### Water–Lithium Correlations

Lithium–water PDFs are characterized by a strong peak centered at ca. 1.9 Å (Figure 6b). Over the probed salt concentration range, the lithium cation tends to maintain water coordination (Figure S-6b) and the resulting water molecules surrounding lithium will organize with a mutual reciprocal distance of ca. 3.2 Å (see above). Such an interaction will strongly affect water organization that, accordingly, shows drastic evolution, as mentioned above. In Figure S-10, we show the coordination number distributions and the average coordination numbers of water hydrogens, Hw, and lithium cations surrounding a reference water oxygen, Ow. Clearly, there is a competition between the two species in solvating Ow. The number of Hw surrounding Ow decreases from 1.7 in neat water down to ca. 0.1 at *c* = 20 m; conversely, lithium progressively increases its solvation number up to 1 ion at *c* = 20 m, reflecting the change in coordination of water and the strong ability of lithium ions to coordinate water.

### Water–Anion Correlations

Water also interacts with the sulfamide portion of the anion via hydrogen-bonding interactions. Water hydrogen–anion oxygen (Hw-OT) and water hydrogen–water oxygen (Hw-Ow) correlations are both influenced by the change in the salt content. Figure 7c,d shows the evolution of corresponding PDFs. Both PDFs are characterized by a distinct peak at 1.8 and 2.0 Å, respectively. The H-bonds involving either Ow or OT as acceptor ones are characterized by a short Hw···Ox distance and a rather linear geometry (Ow–Hw···Ox > 150° (for Ox = Ow and OT) (data not shown)). By integrating the above-mentioned PDFs, one notices that upon increasing the salt content, the number of Ow coordinating Hw decreases from 1 to less than 0.1, while an increase of the number of OT coordinating each Hw is found up to 0.6 at *c* = 20 m concentration (see Figure S-11). Overall, the oxygen coordination (whatever its origin, either water or anion) toward water hydrogen decreases from ca. 1 down to 0.7, reflecting a substantial change in water coordination organization. As reflected by Figure 7e, the anion nitrogen is strongly hindered from the interaction with water by the bulky SO_2_ groups; accordingly, the anion interacts with water only through its OT atoms.

**Figure 8:**
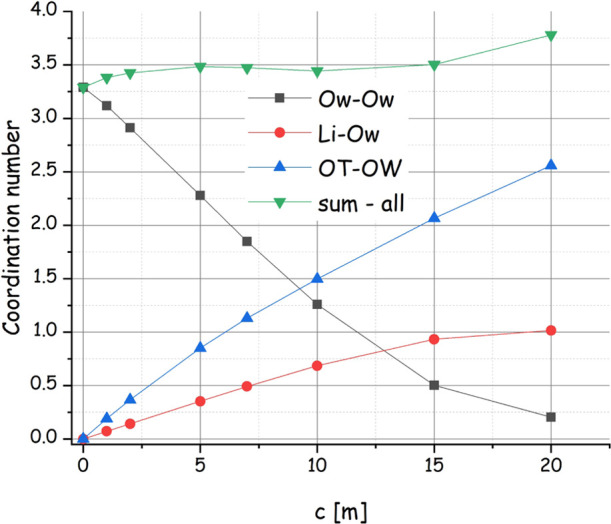
Salt content dependence of the coordination number of water’s oxygen, lithium, and anion oxygen around a reference water oxygen, as obtained by the MD simulations of the LiIM14–H_2_O system.

Overall, upon increasing the salt content, the water solvation environment dramatically changes. Figure 8 shows the composition of the surrounding environment around a reference water oxygen, decomposing it into water molecules (bound to the reference one either via HB-donor or HB-acceptor interactions), lithium cations, and anion’s oxygen atoms. Upon increasing the salt content, the number of water molecules decreases down to a minimum value of 0.25, and, correspondingly, one observes an increase of lithium (up to one ion) and anion oxygen (up to 2.5) solvation of the reference water molecule. The sum of the solvating moieties remains pretty much constant to ca. 3.5. In this scenario, the number of coordinating water molecules drastically decreases not only as a consequence of the smaller water content but also due to their replacement by either lithium or anion oxygen. Accordingly, the ability of the HB acceptor toward water is essentially lost (a negligible amount of Hw approaching the reference Ow), and the ability of the HB donor drastically decreases and involves anion OT rather than Ow.

### Ionic Species Correlations

Despite the strong interaction between lithium and water, the sulfamide moiety of the anion is a competitor with Ow toward lithium coordination. Figure 6d shows the evolution of Li-anion correlations upon increasing the salt content: dilute solutions are characterized by a PDF with an amplitude below one, over more than 10 Å, indicating that the ions are on average fully solvated by water and a very limited amount of contact ion pairs (CIP) exists. Already at concentrations as high as *c* = 5 m, however, a peak manifests at ca. 6.5 Å and its position and amplitude evolve with the increasing salt content. In particular, the Li-anion mutual distance progressively decreases and the number of neighbors increases (Figure S-6d), indicating the development of direct Li-anion correlations: these manifest themselves through the Li–OT interactions and are in competition with Li–Ow correlations. In Figure S-12, we show both the coordination distribution numbers and the average number of either Ow or OT coordinating a reference lithium ion as a function of salt content. It is noticeable that the average number of oxygen atoms (whatever their origin, either water or anion) surrounding the reference lithium ion remains appreciably constant and equal to four. This occurs with a progressive decrease of Ow and an increase of OT belonging to the first lithium solvation shell upon increasing the salt content. It has been noticed previously that even at the highest salt content (*c* = 21 m), a non-negligible fraction of Li(H_2_O)_4_^+^ clusters exists for the case of LiTFSI electrolytes.^13,30^ Here, we observe that also in the case of LiIM14 WiS at *c* = 20 m, ca. 20% of lithium is coordinated by four water molecules with an average coordination number of ca. 2.5 water molecules. Such entities are considered to be fundamental in determining the peculiar conductivity performances of such a class of WiS, allowing lithium ions to diffuse uncoupled from the anions. Consistently, at the same extreme concentration, a fraction of 25% lithium ions appears not to be coordinated by any anion oxygen. There is also a negligible population corresponding to lithium solvation by more than two OT’s. This is at odds with the behavior observed in the case of the TFSI anion:^13^ in particular, we do not find support in the case of the present anion for the rather extreme behavior of lithium that either prefers to be solvated by four water molecules or by four anion oxygens.^13^ Our results indicate an extreme preference of lithium for water coordination rather than anion; presumably, this is due to the different anion sizes that sterically hinder specific interactions in the present case of the IM14 anion.

Lithium–anion nitrogen (NT) correlations also develop but rather as a consequence of the interaction between lithium and the sulfamide moiety than as a direct interaction. To clarify the matter concerning the formation of contact ion pairs (CIP) in alternative to solvent-separated ion pair (SSIP) as a function of salt content, we monitored the probability of the lithium ion to be coordinated by a given number of either anion nitrogen (by a distance of 5 Å) or anion oxygen (by a distance of 2.7 Å). These distributions of coordination numbers are plotted in Figure S-13, together with the corresponding percentages of no-coordination occurrence for the case of nitrogen and oxygen that represent two related evaluations of the fraction of SSIP occurrence. The observed trend recalls the one observed by Suo et al.^13^ for the case of LiTFSI WiS: as it was stressed therein, the reported quantities represent a lower bound estimate of the SSIP. It emerges that the fraction of CIPs (complementary to the SSIP fraction) is very small at low water content, and it increases with the salt amount. Overall, the present results for LiIM14 electrolytes indicate a somehow higher SSIP fraction at the highest concentration than observed for the case of TFSI.^13^ In agreement with the previous discussion, lithium is mainly solvated by water at dilute conditions and a dominant population of SSIP characterizes the ionic species organization. With increasing the salt content, the water solvation around lithium progressively diminishes and a corresponding fraction of CIPs can be appreciated.

Lithium–lithium correlations (Figure 6f) are rather weak, seemingly due to electrostatic reasons, and only at high salt content can one detect the development of a close approach that, however, correspond to very small coordination numbers. Anion–anion correlations (Figure 6e) are also weak. The occurrence of terminal perfluorobutyl −CF_3_ group clustering (Figure 7g) that leads to a constant value of ca. 6 neighbor groups surrounding the reference one, in its first solvation shell, across the whole salt content range is noticeable (Figure S-7g). Such a situation implies the occurrence of anion clustering due to hydrophobic correlations between the fluorous tails, analogously to the case of ionic liquids bearing long fluorinated chains.^51,83−85^

### Mesoscopic Organization

**Figure 9:**
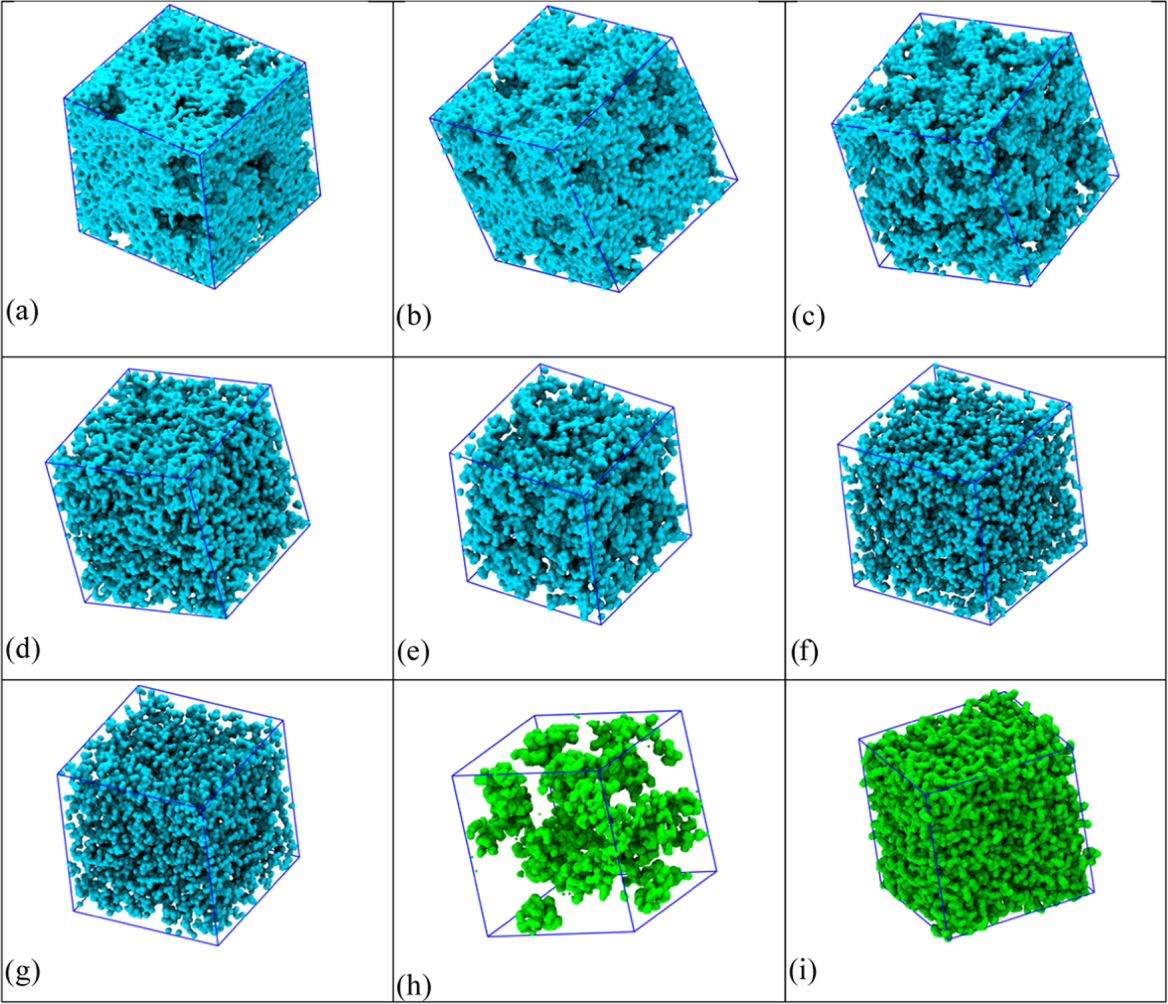
Simulated snapshots of the LiIM14–H_2_O system, where only water is shown ((a–g) for *c* = 1, 2, 5, 7, 10, 15, and 20 m, respectively) and where only the anions are shown ((h, i) for *c* = 1 and 20 m, respectively). Box sizes vary in the range of 85–105 Å.

**Figure 10:**
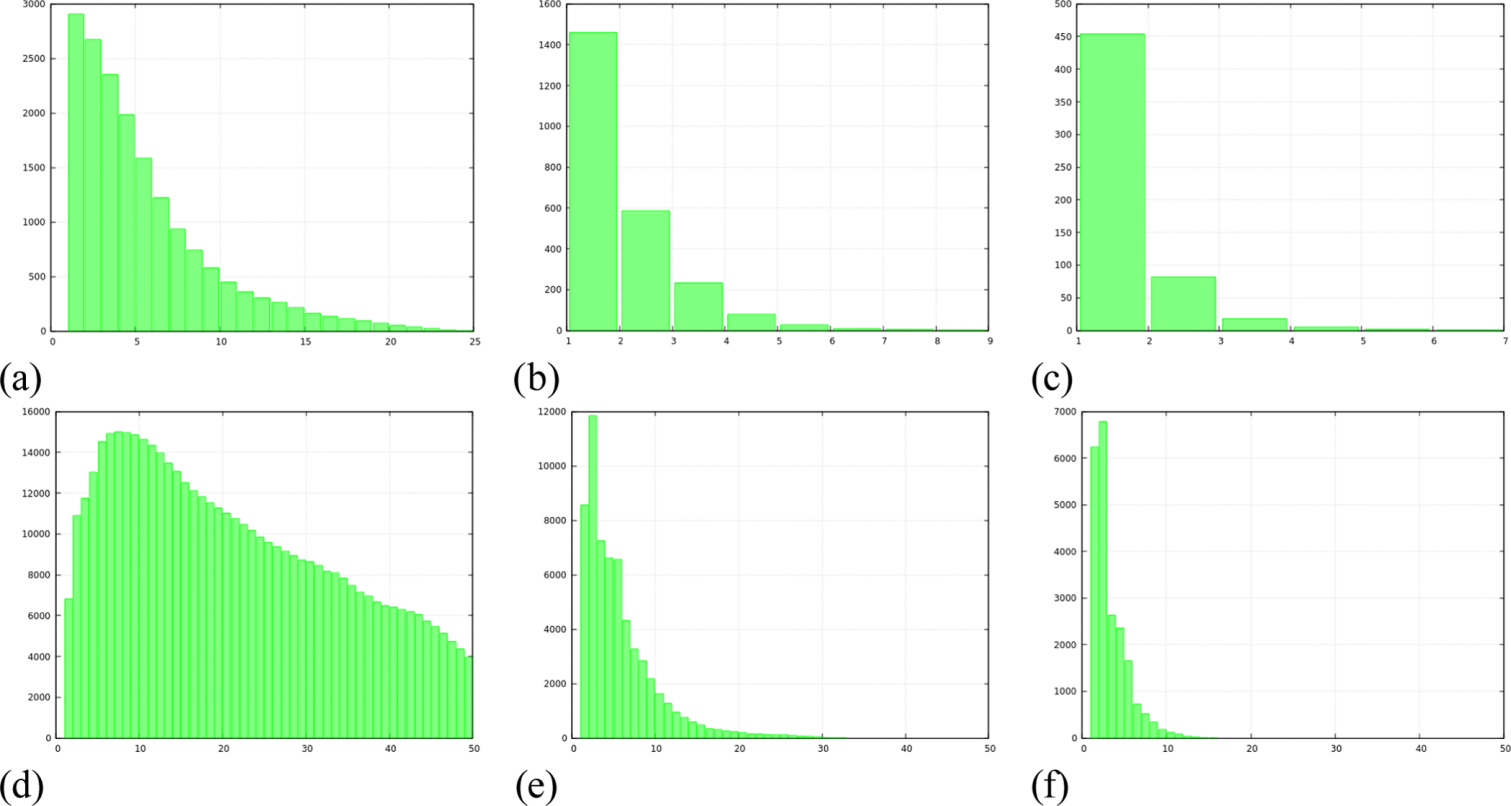
Distribution of chain element numbers for: (top) HB interacting water molecules in LiIM14 WiS, with *c* = (a) 10 m, (b) 15 m, and (c) 20 m and (bottom) HB interacting water molecules and/or Li-Ow interactions in LiIM14 WiS, with *c* = (d) 10 m, (e) 15 m, and (f) 20 m.

Figure 9a–i reports a pictorial view of the simulated boxes after completion of the computations. Therein (Figure 9a–g), only water molecules are shown and empty spaces are filled by anions and lithium ions. The two extreme cases (*c* = 1 and 20 m) are also shown for the complementary case, i.e., showing only the anions and no water/lithium species, for ease of comparison (Figure 9h,i). Inspection of these figures can provide a useful rationalization for several observations done across the manuscript so far. One can appreciate the origin of the peculiarly evident low *Q* features in the X-ray/neutron scattering patterns. In fact, such features are much more intense than reported for the case of the LiTFSI–water system, likely due to the larger size of the fluorinated apolar portion of the anion. At dilute concentrations (between *c* = 1 and 5 m), water constitutes a homogeneous matrix, with a percolating hydrogen-bonding network connecting water molecules. Inside this homogeneous environment, the hydrophobic anions segregate into essentially globular domains, as can be appreciated by comparison of the snapshots reported for the case *c* = 1 m when either only water or only IM14 are plotted (Figure 9a,h, respectively). Ionic species (both Li and IM14) are fully solvated by water, and the fluorinated tails are mutually interacting through dispersive interactions. The increasing salt content leads to the progressive merging of the anion domains, with a high degree of interpenetration of the two micro-segregated phases (Figure 9d,e). At intermediate concentrations, indications of channel-like morphologies built up by water molecules appear, consistently with past observations in the literature.^21^ However, the situation changes further at the highest salt content mixtures, where a finely interpenetrated morphology is observed without evidence of the claimed water channels.^21^ At a high salt content, the anions form a percolating network (Figure 9i) held up by anion–Li and anion–water interactions. As water and lithium are fluorophobic, an efficient anion solvation cannot be achieved and the anions interact with these species mostly through their imide moiety, while the fluorinated moieties remain segregated. Water then efficiently interacts with both lithium and the polar part of the anions, thus creating a finely dispersed aqueous mesh adhering to the anion matrix, with very limited contact with other water molecules. Further indication on the nature of such mesoscopic organization of water molecules in these systems has been obtained by monitoring the spatial extent of chains built up either by hydrogen-bonded water molecules or by a joint interaction between H-bonded water molecules and those connected via a lithium cation. Figure 10a–c,d–f shows such distributions of component numbers for either water–water only or water–water + water–lithium correlated chains, respectively, for the case of the most concentrated solutions.

One detects that dilute solutions (not shown) are characterized by long water-only chains percolating across the simulation box. However, when above *c* = 10 m, the number of HB-connected water molecules drops significantly and the *c* = 20 m system is characterized by just a few (1–2) water molecules that are HB connected to a reference one: no longer chains are appreciable (see Figure 10c). On the other hand, lithium-mediated connections between water molecules show a substantially larger spatial extent. These structures can probably be considered involving those lithium ions that can efficiently migrate and transfer charge across the system.^13,21^ The case of *c* = 10 m shows a very broad distribution for the length of such mixed water–lithium chains; however, the plots for more concentrated solutions prompt that no clear indication of percolating channels that might be responsible for lithium flow in salt-rich mixtures appears. These chains are constituted by max. 20 or even <10 members (for *c* = 15 and 20 m, respectively), which is too small a number to guarantee percolation effects. Overall, the comparison between Figure 4 and Figure 9a–i and similar plots from related papers indicates that dilute solutions are characterized by a strong low *Q* scattering peak that is due to the formation of globular entities formed by segregated anions into the water matrix. They are very large: typically 1–3 nm. Upon increasing the salt content, local electroneutrality and increasing fluorous tail content lead to a progressive merging of these globules into a three-dimensional matrix that eventually, at the highest concentrations, will percolate across the simulation box. Our present results suggest that the low *Q* peak is the fingerprint of alternating anion and water domains, as the *S*(*Q*) decomposition into different contributions leads to water–water and anion–anion peaks out of phase with water–anion anti-peaks (data not shown).^22,28^ This clearly shows that such a low *Q* (X-ray or neutron scattering) peak feature appears at any concentration conditions (similar to what was reported by Zhang et al.^28^ and by Liu et al.,^55^ but at odds with what Tan et al.^22^ claimed). Such a low *Q* feature then represents the signature of a structural organization that is persistent in the WiS system over the whole concentration regime. At low salt content, it clearly reflects the existence of the globular aggregates dispersed in water. When the salt content increases, the peak fingerprints the existence of a distinctly bicontinuous, sponge-like morphology, with mutually excluding domains formed by the more extended phase (water or, at high salt content, anions), which alternates, over nm scale, with the minority one (anions or, at high salt content, water). At concentration extremes (either water-rich or salt-rich conditions), the majority phase constitutes a percolating network hold up either by hydrogen-bonding correlations between water molecules (water-rich case) or by cation/anion and fluorophilic dispersive correlations (salt-rich case). These dominating matrixes are intercalated either by anion globules (water-rich case) or by water–lithium wires (salt-rich case) with extensions of 10–30 Å, but not percolating.

**Figure 11:**
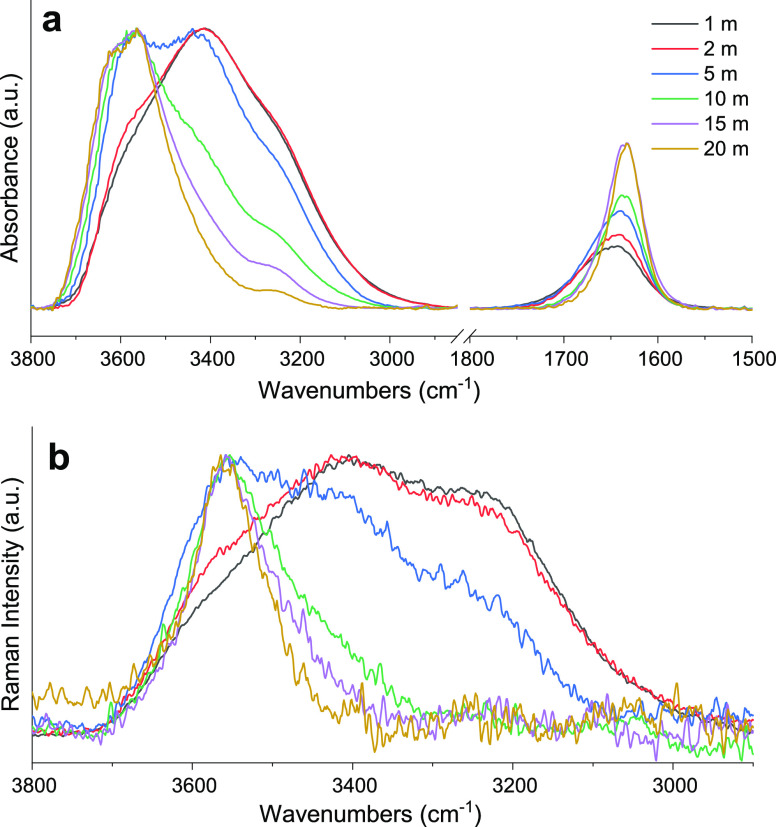
FTIR spectra (a) and Raman spectra (b) acquired at room temperature for LiIM14–H_2_O mixtures at different molalities (*m*). In (b), wavenumbers of the *x*-axis identify Raman shifts.

**Figure 12:**
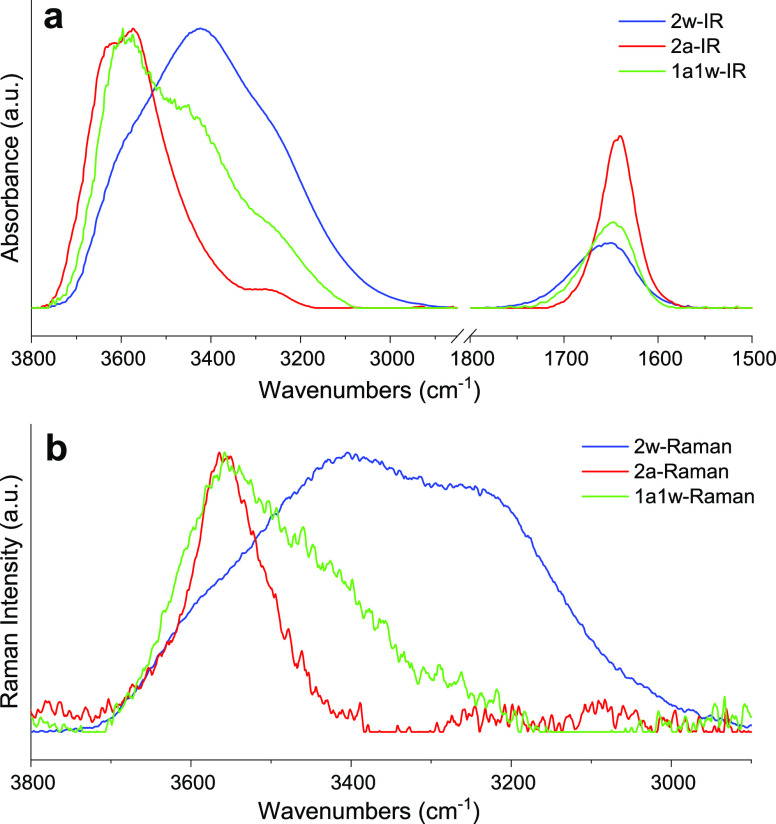
Spectral profiles of the 2w (blue line), 2a (red line), and 1a1w (green line) water populations calculated by MCR-ALS for the (a) FTIR and (b) Raman data sets. In (b), the wavenumbers of the *x*-axis identify the Raman shifts.

To obtain further experimental evidence about intermolecular interactions existing in the LiIM14–water system, vibrational modes of water were analyzed by means of FTIR and Raman spectroscopy. Indeed, the water stretching and bending modes are known to be powerful probes for monitoring the strength and configuration of the H-bond network.^86^ Selected portions of FTIR and Raman spectra for six LiIM14–H_2_O mixtures at *c* = 1, 2, 5, 10, 15, and 20 m are shown in Figure 11a,b, respectively. The water stretching and bending region for FTIR spectroscopy (3800–2850 and 1850–1450 cm^–1^, respectively), as well as the stretching region for Raman spectroscopy (3800–2800 cm^–1^), have been analyzed by means of the MCR-ALS model (see Experimental and Computational Details section). For both the acquired data sets, the spectral evolution as a function of salt concentration has been modeled in terms of a linear combination of three spectral components, each one assigned to water populations with different degrees of intermolecular interactions. In analogy to other spectral decomposition techniques applied to similar systems,^28^ the three spectral profiles for the FTIR and Raman are shown in Figure 12a,b, respectively. For both FTIR and Raman spectral profile sets, a first component, defined as 2w, predominates at the lowest salt concentration (blue lines in Figure 12a,b). In both cases, a great similarity to neat water spectra is observed; these spectral profiles, indeed, are assigned to a bulk-water population, where each water molecule donates on average two H-bond to other water molecules. In particular, in the OH stretching spectral region, the FTIR absorption shows a broad band centered at 3422 cm^–1^, whereas in the Raman profile, three main spectral contributions are distinguishable although overlapped. They are centered at 3560, 3450, and 3260 cm^–1^ and assigned to the OH vibrations of water molecules with an increasing degree of connectivity, respectively.^87^ On the other hand, a second spectral profile, indicated as 2a (red lines in Figure 12a,b), predominates at high LiIM14 concentrations; it is assigned to water molecules that donate two H-bonds to other molecular species, i.e., the IM14 anion. In fact, for both the FTIR and the Raman case, it shows the characteristic spectral features of isolated water in the solution, such as the blue-shifted OH stretching, with its asymmetric and symmetric intramolecular coupling modes falling at 3640 and 3570 cm^–1^, respectively.^88^ Finally, a third spectral component, defined as 1w1a (green lines in Figure 12a,b), is assigned to those water molecules donating on average one H-bond to water; meanwhile, the other is weakly bonded to the IM14 anion. Spectral features, such as bandwidths and positions of the 1a1w spectrum, are intermediate between the 2a and 2w components. Moreover, in the FTIR spectral profiles, the OH bending mode at 1652 cm^–1^ is clearly observable. It is broad in the 2w-IR component, with a full width at half maximum (FWHM) = 88 cm^–1^ and red shifts and distinctly sharpens (down to a FWHM = 45 cm^–1^, in the 2a-IR) with increasing salt concentration. This sharpening is additional evidence of the lack of the H-bonded water network, due to the absence of the intermolecular mode coupling between the pure H–O–H bending and the libration mode, typical of tetrahedral water clusters.^89^

**Figure 13:**
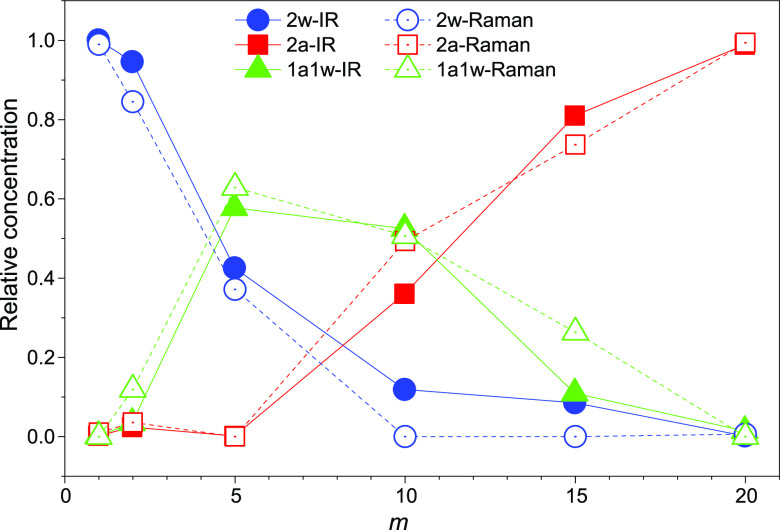
Concentration profiles of the 2w (blue circles), 2a (red squares), and 1a1w (green triangles) water populations calculated by MCR-ALS for the FTIR (filled symbols) and Raman (open symbols) spectral data sets.

The application of the MCR-ALS algorithm to the modeling of experimental FTIR and Raman spectroscopy data delivers the concentration dependence of the three components 2w, 2a, and 1w1a, i.e., the weights of the three water populations 2a, 2w, and 1a1w in building up the observed spectra. Figure 13 displays these relative weights (sum of the three species populations normalized to one), independently calculated for IR and Raman results, as a function of salt concentration. It is noteworthy that, although the two complementary vibrational techniques, FTIR and Raman, experimentally deliver different spectra (Figure 11), the MCR-ALS calculated concentration profiles (Figure 12) show very consistent trends (Figure 13). This strongly supports the reliability of the analysis and prompts for the following interpretative model.

At low salt content, e.g., *c* < 5 m, water is mostly in the 2w form with a small population of 1a1w due to the large abundance of bulk water with respect to the solvation shell of the salt ions (Figure 9a,b). At *c* = 5 m, a distinct increase of the 1a1w water population is observed, up to about 60% of the total, at the expense of 2w water; meanwhile, the 2a water is still absent (Figure 9c). An onset of a structural transition from a water continuous matrix to a bicontinuous water–LiIM14 phase is observed at about *c* = 7 m (Figure 9d). At this concentration, most water molecules donate one H-bond to an adjacent water molecule and the other to the IM14 anion, but still, water clusters survive, contributing to the 2w concentration.

By further increasing the salt concentration (*c* ≥ 10 m), the 2w water population becomes irrelevant; meanwhile, a large increase of the 2a population is highlighted up to almost 100% at *c* = 20 m, at the expense of the 1a1w water. This trend agrees with the findings from MD simulations: upon increasing the salt content, water molecules organize into progressively smaller clusters/chains in the anion matrix. At *c* = 20 m, only a very few water molecules interact with each other, although the average distance between their mass center is 3.2 Å (Figure 6a). They are involved in hydrogen bonds with the anion and coordinated around the cation (Figure 8). This behavior is different from the one observed in the case of the smaller anion TFSI, where a 50% population of 1w1a coordination managed to survive at *c*_TFSI_ = 20 m;^28^ here, presumably due to the much larger hydrophobic portion, the anion network tends to strongly separate water molecules from each other.

## Conclusions

Water-in-salt systems are attracting great attention as appealing electrolytes for energy storage devices. While several WiS have been proposed aiming at extending the electrochemical and liquid state stability, nevertheless, structural investigations have focused mostly on LiTFSI-based WiS systems. Here, we reported the first investigation on the phase diagram, electrochemical properties, structure, and vibrational features of a novel lithium electrolyte based on the ((trifluoromethane)(nonafluorobutane)-sulfonyl)imide anion, a highly asymmetric ionic species. This electrolyte class shows appealing liquid and electrochemical stability windows, with *c* = 20 m mixtures melting at <25 °C, and interesting conductivity performances. The synergic exploitation of IR and Raman spectroscopies together with X-ray small- and wide-angle scattering and molecular dynamics simulations allows achieving a very detailed insight into the structural features of such a system. The liquid state is characterized by a strong segregation between water and hydrophobic fluorous ionic moieties: the large fluorous tails enhance such a segregation as compared, e.g., to the well-known LiTFSI–water systems. In this scenario, dilute solutions are characterized by a peculiar globular organization of anions that are immersed into homogenous bulk-like water. This behavior manifests itself as a very strong X-ray scattering feature, whose presence is ubiquitous across the explored concentration range. Ionic species are fully water solvated, and no ion pairing can be observed. Upon increasing the salt content, lithium keeps on drawing water molecules but begins interacting with the anions by maintaining a constant coordination number of oxygen atoms (either from water or from the anions) over the whole concentration range. Anions tend to be locally neutralized by (water-bearing) lithium ions; otherwise, they interact either with water (via HB interactions) or between themselves through dispersive, fluorophilic interactions, leading to a progressively more and more extended and percolating fluorous matrix. At the highest concentration conditions, the large anion size leads to an extreme fragmentation of a bulk-water hydrogen-bonding network and no more water–water correlations can be detected. Water then organizes in a wire-like manner with a negligible amount of water–water hydrogen-bonding-mediated correlations, but with intermediate lithium ions mediating the correlation and locally neutralizing the system. Overall, the present scenario supports and, considering the different anions, reinforces the proposal that no water channels exist at high salt content. The fluorous matrix percolates across the bulk and is locally intercalated by short water–lithium wires, across which, presumably, lithium hopping occurs. Accordingly, lithium’s peculiar transport properties in WiS systems seem to be related to two concomitant effects, namely: (i) the extreme nanoscale separation between anion-rich and water-rich domains that appear ubiquitous across the probed concentration range and (ii) the existence of water wires across the anion matrix where a large population of Li(H_2_O)_4_^+^ clusters, fully disentangled from anion coordination, would diffuse at an enhanced rate, as compared to anion-bound lithium.

Furthermore, we stress that while these preliminary characterizations indicate that the anodic stability of the LiIM14 electrolyte does not match the requirements for high voltage (>4 V) battery systems, this electrolyte still represents a new, appealing way for enhancing the electrochemical robustness of aqueous solutions toward oxidation. For instance, concentrated aqueous electrolytes based on the IM14 anion might find applications in metal–oxygen batteries (i.e., Zn/O_2_ systems operate below 2 V) and, upon an enhancement of the anodic stability of even a few hundreds of mV, in Li/S post-lithium batteries.

The present study provides a detailed characterization of the organization in an evolved WiS (as compared to TFSI based ones), suggesting a role for strong anion asymmetry in structural organization and thus prompting for knowledge-oriented modifications to be applied to the existing WiS systems.

## Supporting Information Available

Supporting figures on the density of LiIM14–H_2_O mixtures as a function of temperature; log–log version of SAXS data; computed neutron scattering patterns; comparison between the experimental and computed density at 25 °C; MD-derived running coordination numbers from *g*(*r*)’s; several solvation distribution functions and corresponding average coordination numbers (PDF)

## References

[ref1] Suo L.; Borodin O.; Gao T.; Olguin M.; Ho J.; Fan X.; Luo C.; Wang C.; Xu K. Water-in-Salt” Electrolyte Enables High-Voltage Aqueous Lithium-Ion Chemistries. Science 2015, 350, 938–943. 10.1126/science.aab1595. 26586759

[ref2] Liang T.; Hou R.; Dou Q.; Zhang H.; Yan X. The Applications of Water-in-Salt Electrolytes in Electrochemical Energy Storage Devices. Adv. Funct. Mater. 2021, 31, 2006749 10.1002/adfm.202006749.

[ref3] Yamada Y.; Wang J.; Ko S.; Watanabe E.; Yamada A. Advances and Issues in Developing Salt-Concentrated Battery Electrolytes. Nat. Energy 2019, 4, 269–280. 10.1038/s41560-019-0336-z.

[ref4] Azov V. A.; Egorova K. S.; Seitkalieva M. M.; Kashin A. S.; Ananikov V. P. “Solvent-in-Salt” Systems for Design of New Materials in Chemistry, Biology and Energy Research. Chem. Soc. Rev. 2018, 47, 1250–1284. 10.1039/c7cs00547d. 29410995

[ref5] Zheng Q.; Miura S.; Miyazaki K.; Ko S.; Watanabe E.; Okoshi M.; Chou C. P.; Nishimura Y.; Nakai H.; Kamiya T.; Honda T.; Akikusa J.; Yamada Y.; Yamada A. Sodium- and Potassium-Hydrate Melts Containing Asymmetric Imide Anions for High-Voltage Aqueous Batteries. Angew. Chem., Int. Ed. 2019, 58, 14202–14207. 10.1002/anie.201908830. 31359550

[ref6] Becker M.; Kühnel R. S.; Battaglia C. Water-in-Salt Electrolytes for Aqueous Lithium-Ion Batteries with Liquidus Temperatures below −10 °c. Chem. Commun. 2019, 55, 12032–12035. 10.1039/c9cc04495g. 31531496

[ref7] Reber D.; Kühnel R. S.; Battaglia C. High-Voltage Aqueous Supercapacitors Based on NaTFSI. Sustainable Energy Fuels 2017, 1, 2155–2161. 10.1039/c7se00423k.

[ref8] Amiri M.; Bélanger D. Physicochemical and Electrochemical Properties of Water-in-Salt Electrolytes. ChemSusChem 2021, 14, 2487–2500. 10.1002/cssc.202100550. 33973406

[ref9] Ko S.; Yamada Y.; Miyazaki K.; Shimada T.; Watanabe E.; Tateyama Y.; Kamiya T.; Honda T.; Akikusa J.; Yamada A. Lithium-Salt Monohydrate Melt: A Stable Electrolyte for Aqueous Lithium-Ion Batteries. Electrochem. Commun. 2019, 104, 106488 10.1016/j.elecom.2019.106488.

[ref10] Dubouis N.; Lemaire P.; Mirvaux B.; Salager E.; Deschamps M.; Grimaud A. The Role of the Hydrogen Evolution Reaction in the Solid-Electrolyte Interphase Formation Mechanism for “Water-in-Salt” Electrolytes. Energy Environ. Sci. 2018, 11, 3491–3499. 10.1039/c8ee02456a.

[ref11] Shen Y.; Liu B.; Liu X.; Liu J.; Ding J.; Zhong C.; Hu W. Water-in-Salt Electrolyte for Safe and High-Energy Aqueous Battery. Energy Storage Mater. 2021, 34, 461–474. 10.1016/j.ensm.2020.10.011.

[ref12] Yamada Y.; Usui K.; Sodeyama K.; Ko S.; Tateyama Y.; Yamada A. Hydrate-Melt Electrolytes for High-Energy-Density Aqueous Batteries. Nat. Energy 2016, 1, 16129 10.1038/nenergy.2016.129.

[ref13] Borodin O.; Suo L.; Gobet M.; Ren X.; Wang F.; Faraone A.; Peng J.; Olguin M.; Schroeder M.; Ding M. S.; Gobrogge E.; von Wald Cresce A.; Munoz S.; Dura J. A.; Greenbaum S.; Wang C.; Xu K. Liquid Structure with Nano-Heterogeneity Promotes Cationic Transport in Concentrated Electrolytes. ACS Nano 2017, 11, 10462–10471. 10.1021/acsnano.7b05664. 29016112

[ref14] Suo L.; Borodin O.; Wang Y.; Rong X.; Sun W.; Fan X.; Xu S.; Schroeder M. A.; Cresce A. V.; Wang F.; Yang C.; Hu Y. S.; Xu K.; Wang C. Water-in-Salt” Electrolyte Makes Aqueous Sodium-Ion Battery Safe, Green, and Long-Lasting. Adv. Energy Mater. 2017, 7, 1701189 10.1002/aenm.201701189.

[ref15] Chen M.; Feng G.; Qiao R. Water-in-Salt Electrolytes: An Interfacial Perspective. Curr. Opin. Colloid Interface Sci. 2020, 47, 99–110. 10.1016/j.cocis.2019.12.011.

[ref16] Li H.; Kurihara T.; Yang D.; Watanabe M.; Ishihara T. A Novel Aqueous Dual-Ion Battery Using Concentrated Bisalt Electrolyte. Energy Storage Mater. 2021, 38, 454–461. 10.1016/j.ensm.2021.03.029.

[ref17] Miyazaki K.; Takenaka N.; Watanabe E.; Iizuka S.; Yamada Y.; Tateyama Y.; Yamada A. First-Principles Study on the Peculiar Water Environment in a Hydrate-Melt Electrolyte. J. Phys. Chem. Lett. 2019, 10, 6301–6305. 10.1021/acs.jpclett.9b02207. 31512877

[ref18] Ding M. S.; Xu K. Phase Diagram, Conductivity, and Glass Transition of LiTFSI-H2O Binary Electrolytes. J. Phys. Chem. C 2018, 122, 16624–16629. 10.1021/acs.jpcc.8b05193.

[ref19] Ding M. S.; Von Cresce A.; Xu K. Conductivity, Viscosity, and Their Correlation of a Super-Concentrated Aqueous Electrolyte. J. Phys. Chem. C 2017, 121, 2149–2153. 10.1021/acs.jpcc.6b12636.

[ref20] Sakamoto R.; Yamashita M.; Nakamoto K.; Zhou Y.; Yoshimoto N.; Fujii K.; Yamaguchi T.; Kitajou A.; Okada S. Local Structure of a Highly Concentrated NaClO4aqueous Solution-Type Electrolyte for Sodium Ion Batteries. Phys. Chem. Chem. Phys. 2020, 22, 26452–26458. 10.1039/d0cp04376a. 33180893

[ref21] Lim J.; Park K.; Lee H.; Kim J.; Kwak K.; Cho M. Nanometric Water Channels in Water-in-Salt Lithium Ion Battery Electrolyte. J. Am. Chem. Soc. 2018, 140, 15661–15667. 10.1021/jacs.8b07696. 30358996

[ref22] Tan P.; Yue J.; Yu Y.; Liu B.; Liu T.; Zheng L.; He L.; Zhang X.; Suo L.; Hong L. Solid-Like Nano-Anion Cluster Constructs a Free Lithium-Ion-Conducting Superfluid Framework in a Water-in-Salt Electrolyte. J. Phys. Chem. C 2021, 125, 11838–11847. 10.1021/acs.jpcc.1c01663.

[ref23] González M. A.; Borodin O.; Kofu M.; Shibata K.; Yamada T.; Yamamuro O.; Xu K.; Price D. L.; Saboungi M.-L. Nanoscale Relaxation in “Water-in-Salt” and “Water-in-Bisalt” Electrolytes. J. Phys. Chem. Lett. 2020, 7279–7284. 10.1021/acs.jpclett.0c01765. 32787289

[ref24] Popov I.; Sacci R. L.; Sanders N. C.; Matsumoto R. A.; Thompson M. W.; Osti N. C.; Kobayashi T.; Tyagi M.; Mamontov E.; Pruski M.; Cummings P. T.; Sokolov A. P. Critical Role of Anion-Solvent Interactions for Dynamics of Solvent-in-Salt Solutions. J. Phys. Chem. C 2020, 124, 8457–8466. 10.1021/acs.jpcc.9b10807.

[ref25] Zhang M.; Hao H.; Zhou D.; Duan Y.; Wang Y.; Bian H. Understanding the Microscopic Structure of a “Water-in-Salt” Lithium Ion Battery Electrolyte Probed with Ultrafast IR Spectroscopy. J. Phys. Chem. C 2020, 124, 8594–8604. 10.1021/acs.jpcc.0c00937.

[ref26] Reber D.; Takenaka N.; Kühnel R. S.; Yamada A.; Battaglia C. Impact of Anion Asymmetry on Local Structure and Supercooling Behavior of Water-in-Salt Electrolytes. J. Phys. Chem. Lett. 2020, 11, 4720–4725. 10.1021/acs.jpclett.0c00806. 32492350

[ref27] Jeon J.; Lee H.; Choi J. H.; Cho M. Modeling and Simulation of Concentrated Aqueous Solutions of LiTFSI for Battery Applications. J. Phys. Chem. C 2020, 124, 11790–11799. 10.1021/acs.jpcc.0c02187.

[ref28] Zhang Y.; Lewis N. H. C.; Mars J.; Wan G.; Weadock N. J.; Takacs C. J.; Lukatskaya M. R.; Steinrück H.-G.; Toney M. F.; Tokmakoff A.; Maginn E. J. Water-in-Salt LiTFSI Aqueous Electrolytes. 1. Liquid Structure from Combined Molecular Dynamics Simulation and Experimental Studies. J. Phys. Chem. B 2021, 125, 4501–4513. 10.1021/acs.jpcb.1c02189. 33904299

[ref29] Biswas A.; Mallik B. S. Ultrafast Aqueous Dynamics in Concentrated Electrolytic Solutions of Lithium Salt and Ionic Liquid. J. Phys. Chem. B 2020, 124, 9898–9912. 10.1021/acs.jpcb.0c06221. 33105991

[ref30] Han K. S.; Yu Z.; Wang H.; Redfern P. C.; Ma L.; Cheng L.; Chen Y.; Hu J. Z.; Curtiss L. A.; Xu K.; Murugesan V.; Mueller K. T. Origin of Unusual Acidity and Li+Diffusivity in a Series of Water-in-Salt Electrolytes. J. Phys. Chem. B 2020, 124, 5284–5291. 10.1021/acs.jpcb.0c02483. 32484675

[ref31] Mendez-Morales T.; Li Z.; Salanne M. Computational Screening of the Physical Properties of Water-in-Salt Electrolytes. Batteries Supercaps 2021, 4, 646–652. 10.1002/batt.202000237.

[ref32] Borodin O.; Self J.; Persson K. A.; Wang C.; Xu K. Uncharted Waters: Super-Concentrated Electrolytes. Joule 2020, 4, 69–100. 10.1016/j.joule.2019.12.007.

[ref33] Yu Z.; Curtiss L. A.; Winans R. E.; Zhang Y.; Li T.; Cheng L. Asymmetric Composition of Ionic Aggregates and the Origin of High Correlated Transference Number in Water-in-Salt Electrolytes. J. Phys. Chem. Lett. 2020, 11, 1276–1281. 10.1021/acs.jpclett.9b03495. 31951143

[ref34] Forero-Saboya J.; Hosseini-Bab-Anari E.; Abdelhamid M. E.; Moth-Poulsen K.; Johansson P. Water-in-Bisalt Electrolyte with Record Salt Concentration and Widened Electrochemical Stability Window. J. Phys. Chem. Lett. 2019, 10, 4942–4946. 10.1021/acs.jpclett.9b01467. 31403300

[ref35] Tsurumura T.; Hashimoto Y.; Morita M.; Umebayashi Y.; Fujii K. Anion Coordination Characteristics of Ion-Pair Complexes in Highly Concentrated Aqueous Lithium Bis(Trifluoromethanesulfonyl) Amide Electrolytes. Anal. Sci. 2019, 35, 289–294. 10.2116/analsci.18P407. 30393238

[ref36] Horwitz G.; Rodríguez C. R.; Steinberg P. Y.; Burton G.; Corti H. R. Mobility-Viscosity Decoupling and Cation Transport in Water-in-Salt Lithium Electrolytes. Electrochim. Acta 2020, 359, 136915 10.1016/j.electacta.2020.136915.

[ref37] Kühnel R.-S.; Reber D.; Battaglia C. Perspective—Electrochemical Stability of Water-in-Salt Electrolytes. J. Electrochem. Soc. 2020, 167, 070544 10.1149/1945-7111/ab7c6f.

[ref38] Droguet L.; Grimaud A.; Fontaine O.; Tarascon J. M. Water-in-Salt Electrolyte (WiSE) for Aqueous Batteries: A Long Way to Practicality. Adv. Energy Mater. 2020, 10, 2002440 10.1002/aenm.202002440.

[ref39] Tan J.; Liu J. Electrolyte Engineering Toward High-Voltage Aqueous Energy Storage Devices. Energy Environ. Mater. 2020, 4, 302–306. 10.1002/eem2.12125.

[ref40] Reber D.; Grissa R.; Becker M.; Kühnel R.; Battaglia C. Anion Selection Criteria for Water-in-Salt Electrolytes. Adv. Energy Mater. 2021, 11, 2002913 10.1002/aenm.202002913.

[ref41] von Wald Cresce A.; Xu K. Aqueous Lithium-ion Batteries. Carbon Energy 2021, 3, 721–751. 10.1002/cey2.106.

[ref42] Watanabe H.; Arai N.; Nozaki E.; Han J.; Fujii K.; Ikeda K.; Otomo T.; Ueno K.; Dokko K.; Watanabe M.; Kameda Y.; Umebayashi Y. Local Structure of Li + in Superconcentrated Aqueous LiTFSA Solutions. J. Phys. Chem. B 2021, 125, 7477–7484. 10.1021/acs.jpcb.1c04693. 34196549

[ref43] Marcus Y. Unconventional Deep Eutectic Solvents: Aqueous Salt Hydrates. ACS Sustainable Chem. Eng. 2017, 5, 11780–11787. 10.1021/acssuschemeng.7b03528.

[ref44] Lux S. F.; Terborg L.; Hachmöller O.; Placke T.; Meyer H.-W.; Passerini S.; Winter M.; Nowak S. LiTFSI Stability in Water and Its Possible Use in Aqueous Lithium-Ion Batteries: PH Dependency, Electrochemical Window and Temperature Stability. J. Electrochem. Soc. 2013, 160, A1694–A1700. 10.1149/2.039310jes.

[ref45] Reber D.; Kühnel R. S.; Battaglia C. Suppressing Crystallization of Water-in-Salt Electrolytes by Asymmetric Anions Enables Low-Temperature Operation of High-Voltage Aqueous Batteries. ACS Mater. Lett. 2019, 1, 44–51. 10.1021/acsmaterialslett.9b00043.

[ref46] Ko S.; Yamada Y.; Yamada A. A 62 m K-Ion Aqueous Electrolyte. Electrochem. Commun. 2020, 116, 106764 10.1016/j.elecom.2020.106764.

[ref47] Thareja S.; Kumar A. “water-In-Salt” Electrolyte-Based High-Voltage (2.7 V) Sustainable Symmetric Supercapacitor with Superb Electrochemical Performance - An Analysis of the Role of Electrolytic Ions in Extending the Cell Voltage. ACS Sustainable Chem. Eng. 2021, 9, 2338–2347. 10.1021/acssuschemeng.0c08604.

[ref48] Horwitz G.; Härk E.; Steinberg P. Y.; Cavalcanti L. P.; Risse S.; Corti H. R. The Nanostructure of Water-in-Salt Electrolytes Revisited: Effect of the Anion Size. ACS Nano 2021, 15, 11564–11572. 10.1021/acsnano.1c01737. 34255484

[ref49] Horwitz G.; Steinberg P. Y.; Corti H. R. Volumetric and Viscosity Properties of Water-in-Salt Lithium Electrolytes: A Comparison with Ionic Liquids and Hydrated Molten Salts. J. Chem. Thermodyn. 2021, 158, 106457 10.1016/j.jct.2021.106457.

[ref50] Russina O.; Triolo A. New Experimental Evidence Supporting the Mesoscopic Segregation Model in Room Temperature Ionic Liquids. Faraday Discuss. 2012, 154, 97–109. 10.1039/C1FD00073J. 22455016

[ref51] Lo Celso F.; Appetecchi G. B.; Simonetti E.; Zhao M.; Jr E. W. C.; Keiderling U.; Gontrani L.; Triolo A. Microscopic Structural and Dynamic Features in Triphilic Room Temperature Ionic Liquids. Front. Chem. 2019, 7, 285 10.3389/fchem.2019.00285. 31119123 PMC6507529

[ref52] Lo Celso F.; Appetecchi G. B.; Jafta C. J.; Gontrani L.; Canongia Lopes J. N.; Triolo A.; Russina O. Nanoscale Organization in the Fluorinated Room Temperature Ionic Liquid: Tetraethyl Ammonium (Trifluoromethanesulfonyl)(Nonafluorobutylsulfonyl)Imide. J. Chem. Phys. 2018, 148, 193816 10.1063/1.5016236. 30307172

[ref53] Lo Celso F.; Appetecchi G. B.; Simonetti E.; Keiderling U.; Gontrani L.; Triolo A.; Russina O. Mesoscopic Structural Organization in Fluorinated Pyrrolidinium-Based Room Temperature Ionic Liquids. J. Mol. Liq. 2019, 289, 111110 10.1016/j.molliq.2019.111110.

[ref54] Triolo A.; Lo Celso F.; Ottaviani C.; Ji P.; Appetecchi G. B.; Leonelli F.; Keeble D. S.; Russina O. Structural Features of Selected Protic Ionic Liquids Based on a Super-Strong Base. Phys. Chem. Chem. Phys. 2019, 21, 25369–25378. 10.1039/c9cp03927a. 31709430

[ref55] Liu X.; Yu Z.; Sarnello E.; Qian K.; Seifert S.; Winans R. E.; Cheng L.; Li T. Microscopic Understanding of the Ionic Networks of “Water-in-Salt” Electrolytes. Energy Mater. Adv. 2021, 2021, 7368420 10.34133/2021/7368420.

[ref56] Filik J.; Ashton A. W.; Chang P. C. Y.; Chater P. A.; Day S. J.; Drakopoulos M.; Gerring M. W.; Hart M. L.; Magdysyuk O. V.; Michalik S.; Smith A.; Tang C. C.; Terrill N. J.; Wharmby M. T.; Wilhelm H. Processing Two-Dimensional X-Ray Diffraction and Small-Angle Scattering Data in DAWN 2. J. Appl. Crystallogr. 2017, 50, 959–966. 10.1107/S1600576717004708. 28656043 PMC5458597

[ref57] Jaumot J.; de Juan A.; Tauler R. MCR-ALS GUI 2.0: New Features and Applications. Chemom. Intell. Lab. Syst. 2015, 140, 1–12. 10.1016/j.chemolab.2014.10.003.

[ref58] Garrido M.; Rius F. X.; Larrechi M. S. Multivariate Curve Resolution-Alternating Least Squares (MCR-ALS) Applied to Spectroscopic Data from Monitoring Chemical Reactions Processes. Anal. Bioanal. Chem. 2008, 390, 2059–2066. 10.1007/s00216-008-1955-6. 18320174

[ref59] Ahmadi R.; Hemmateenejad B.; Safavi A.; Shojaeifard Z.; Shahsavar A.; Mohajeri A.; Heydari Dokoohaki M.; Zolghadr A. R. Deep Eutectic-Water Binary Solvent Associations Investigated by Vibrational Spectroscopy and Chemometrics. Phys. Chem. Chem. Phys. 2018, 20, 18463–18473. 10.1039/c8cp00409a. 29947372

[ref60] Hess B.; Kutzner C.; van der Spoel D.; Lindahl E. GROMACS 4: Algorithms for Highly Efficient, Load-Balanced, and Scalable Molecular Simulation. J. Chem. Theory Comput. 2008, 4, 435–447. 10.1021/ct700301q. 26620784

[ref61] Van Der Spoel D.; Lindahl E.; Hess B.; Groenhof G.; Mark A. E.; Berendsen H. J. C. GROMACS: Fast, Flexible, and Free. J. Comput. Chem. 2005, 26, 1701–1718. 10.1002/jcc.20291. 16211538

[ref62] Lopes J. N. C.; Pádua A. A. H. Molecular Force Field for Ionic Liquids Composed of Triflate or Bistriflylimide Anions. J. Phys. Chem. B 2004, 108, 16893–16898. 10.1021/jp0476545.

[ref63] Gouveia A. S. L.; Bernardes C. E. S.; Tomé L. C.; Lozinskaya E. I.; Vygodskii Y. S.; Shaplov A. S.; Lopes J. N. C.; Marrucho I. M. Ionic Liquids with Anions Based on Fluorosulfonyl Derivatives: From Asymmetrical Substitutions to a Consistent Force Field Model. Phys. Chem. Chem. Phys. 2017, 19, 29617–29624. 10.1039/c7cp06081e. 29083012

[ref64] Shimizu K.; Almantariotis D.; Gomes M. F. C.; Pádua A. A. H.; Canongia Lopes J. N. Molecular Force Field for Ionic Liquids V: Hydroxyethylimidazolium, Dimethoxy-2- Methylimidazolium, and Fluoroalkylimidazolium Cations and Bis(Fluorosulfonyl)Amide, Perfluoroalkanesulfonylamide, and Fluoroalkylfluorophosphate Anions. J. Phys. Chem. B 2010, 114, 3592–3600. 10.1021/jp9120468. 20175555

[ref65] Berendsen H. J. C.; Grigera J. R.; Straatsma T. P. The Missing Term in Effective Pair Potentials. J. Phys. Chem. A 1987, 91, 6269–6271. 10.1021/j100308a038.

[ref66] Dang L. X. Development of Nonadditive Intermolecular Potentials Using Molecular Dynamics: Solvation of Li+ and F- Ions in Polarizable Water. J. Chem. Phys. 1992, 96, 6970–6977. 10.1063/1.462555.

[ref67] Martínez L.; Andrade R.; Birgin E. G.; Martínez J. M. PACKMOL: A Package for Building Initial Configurations for Molecular Dynamics Simulations. J. Comput. Chem. 2009, 30, 2157–2164. 10.1002/jcc.21224. 19229944

[ref68] Bussi G.; Donadio D.; Parrinello M. Canonical Sampling through Velocity Rescaling. J. Chem. Phys. 2007, 126, 014101 10.1063/1.2408420. 17212484

[ref69] Parrinello M.; Rahman A. Polymorphic Transitions in Single Crystals: A New Molecular Dynamics Method. J. Appl. Phys. 1981, 52, 7182–7190. 10.1063/1.328693.

[ref70] Darden T.; York D.; Pedersen L. Particle Mesh Ewald: An N·log(N) Method for Ewald Sums in Large Systems. J. Chem. Phys. 1993, 98, 10089–10092. 10.1063/1.464397.

[ref71] Essmann U.; Perera L.; Berkowitz M. L.; Darden T.; Lee H.; Pedersen L. G. A Smooth Particle Mesh Ewald Method. J. Chem. Phys. 1995, 103, 8577–8593. 10.1063/1.470117.

[ref72] Humphrey W.; Dalke A.; Schulten K. VMD: Visual Molecular Dynamics. J. Mol. Graphics 1996, 14, 33–38. 10.1016/0263-7855(96)00018-5. 8744570

[ref73] Brehm M.; Kirchner B. TRAVIS - A Free Analyzer and Visualizer for Monte Carlo and Molecular Dynamics Trajectories. J. Chem. Inf. Model. 2011, 51, 2007–2023. 10.1021/ci200217w. 21761915

[ref74] Hollóczki O.; Macchiagodena M.; Weber H.; Thomas M.; Brehm M.; Stark A.; Russina O.; Triolo A.; Kirchner B. Triphilic Ionic-Liquid Mixtures: Fluorinated and Non-Fluorinated Aprotic Ionic-Liquid Mixtures. ChemPhysChem 2015, 16, 3325–3333. 10.1002/cphc.201500473. 26305804 PMC4641458

[ref75] Brehm M.; Thomas M.; Gehrke S.; Kirchner B. TRAVIS—A Free Analyzer for Trajectories from Molecular Simulation. J. Chem. Phys. 2020, 152, 164105 10.1063/5.0005078. 32357781

[ref76] Ozkanlar A.; Clark A. E. ChemNetworks: A Complex Network Analysis Tool for Chemical Systems. J. Comput. Chem. 2014, 35, 495–505. 10.1002/jcc.23506. 24311311

[ref77] Vogel H. The Law of the Relation between the Viscosity of Liquids and the Temperature. Phys. Z. 1921, 22, 645.

[ref78] Fulcher G. S. Analysis Of Recent Measurements Of The Viscosity Of Glasses. J. Am. Ceram. Soc. 1925, 8, 789–794. 10.1111/j.1151-2916.1925.tb18582.x.

[ref79] Tammann G.; Hesse W. Die Abhängigkeit Der Viscosität von Der Temperatur Bie Unterkühlten Flüssigkeiten. Z. Anorg. Allg. Chem. 1926, 156, 245–257. 10.1002/zaac.19261560121.

[ref80] Li Z.; Bouchal R.; Mendez-Morales T.; Rollet A. L.; Rizzi C.; Le Vot S.; Favier F.; Rotenberg B.; Borodin O.; Fontaine O.; Salanne M. Transport Properties of Li-TFSI Water-in-Salt Electrolytes. J. Phys. Chem. B 2019, 123, 10514–10521. 10.1021/acs.jpcb.9b08961. 31726827

[ref81] Marinaro M.; Bresser D.; Beyer E.; Faguy P.; Hosoi K.; Li H.; Sakovica J.; Amine K.; Wohlfahrt-Mehrens M.; Passerini S. Bringing Forward the Development of Battery Cells for Automotive Applications: Perspective of R&D Activities in China, Japan, the EU and the USA. J. Power Sources 2020, 459, 228073 10.1016/j.jpowsour.2020.228073.

[ref82] Duboué-Dijon E.; Laage D. Characterization of the Local Structure in Liquid Water by Various Order Parameters. J. Phys. Chem. B 2015, 119, 8406–8418. 10.1021/acs.jpcb.5b02936. 26054933 PMC4516314

[ref83] Russina O.; Lo Celso F.; Di Michiel M.; Passerini S.; Appetecchi G. B.; Castiglione F.; Mele A.; Caminiti R.; Triolo A. Mesoscopic Structural Organization in Triphilic Room Temperature Ionic Liquids. Faraday Discuss. 2014, 167, 499 10.1039/c3fd00056g. 24640508

[ref84] Lo Celso F.; Yoshida Y.; Castiglione F.; Ferro M.; Mele A.; Jafta C. J.; Triolo A.; Russina O. Direct Experimental Observation of Mesoscopic Fluorous Domains in Fluorinated Room Temperature Ionic Liquids. Phys. Chem. Chem. Phys. 2017, 19, 13101–13110. 10.1039/C7CP01971H. 28489101

[ref85] Russina O.; Lo Celso F.; Plechkova N.; Jafta C. J.; Appetecchi G. B.; Triolo A. Mesoscopic Organization in Ionic Liquids. Top. Curr. Chem. 2017, 375, 247–263. 10.1007/978-3-319-89794-3_9. 28516337

[ref86] Seki T.; Chiang K. Y.; Yu C. C.; Yu X.; Okuno M.; Hunger J.; Nagata Y.; Bonn M. The Bending Mode of Water: A Powerful Probe for Hydrogen Bond Structure of Aqueous Systems. J. Phys. Chem. Lett. 2020, 11, 8459–8469. 10.1021/acs.jpclett.0c01259. 32931284 PMC7584361

[ref87] Brubach J. B.; Mermet A.; Filabozzi A.; Gerschel A.; Lairez D.; Krafft M. P.; Roy P. Dependence of Water Dynamics upon Confinement Size. J. Phys. Chem. B 2001, 105, 430–435. 10.1021/jp002983s.

[ref88] Cringus D.; Jansen T. L. C.; Pshenichnikov M. S.; Wiersma D. A. Ultrafast Anisotropy Dynamics of Water Molecules Dissolved in Acetonitrile. J. Chem. Phys. 2007, 127, 084507 10.1063/1.2771178. 17764269

[ref89] Yu C. C.; Chiang K. Y.; Okuno M.; Seki T.; Ohto T.; Yu X.; Korepanov V.; Hamaguchi Ho.; Bonn M.; Hunger J.; Nagata Y. Vibrational Couplings and Energy Transfer Pathways of Water’s Bending Mode. Nat. Commun. 2020, 11, 5977 10.1038/s41467-020-19759-w. 33239630 PMC7688972

