# Supplementary material for: Liquid Structure of a Water-in-Salt Electrolyte with a Remarkably Asymmetric Anion
Source: J Phys Chem B. 2021 Nov 5;125(45):12500–17. doi: 10.1021/acs.jpcb.1c06759 (PMC9282637; doi:10.1021/acs.jpcb.1c06759)
Supplement: Supplementary file 1 — jp1c06759_si_001.pdf [file jp1c06759_si_001.pdf]

Liquid Structure of a Water-In-Salt Electrolyte with a Remarkably Asymmetric Anion.

Alessandro Triolo[1,*], Valerio Di Lisio[2], Fabrizio Lo Celso[1,3], Giovanni B. Appetecchi[4], Barbara Fazio[5], Philip Chater[6], Andrea Martinelli[2], Fabio Sciubba[2,7] and Olga Russina[1,2]

[1] Laboratorio Liquidi Ionici, Istituto Struttura della Materia, Consiglio Nazionale delle Ricerche, (ISM-CNR) Rome, Italy

[2] Department of Chemistry, University of Rome Sapienza, Rome, Italy

[3] Department of Physics and Chemistry, Università di Palermo, Palermo, Italy

[4] ENEA, SSPT-PROMAS-MATPRO Technical Unit, Rome, Italy

[5] Istituto Processi Chimico-Fisici, Consiglio Nazionale delle Ricerche, (IPCF-CNR) Messina, Italy

[6] Diamond House, Harwell Science & Innovation Campus, Diamond Light Source, Ltd., Didcot, UK

[7] NMR-Based Metabolomics Laboratory (NMLab), Sapienza University of Rome, Italy

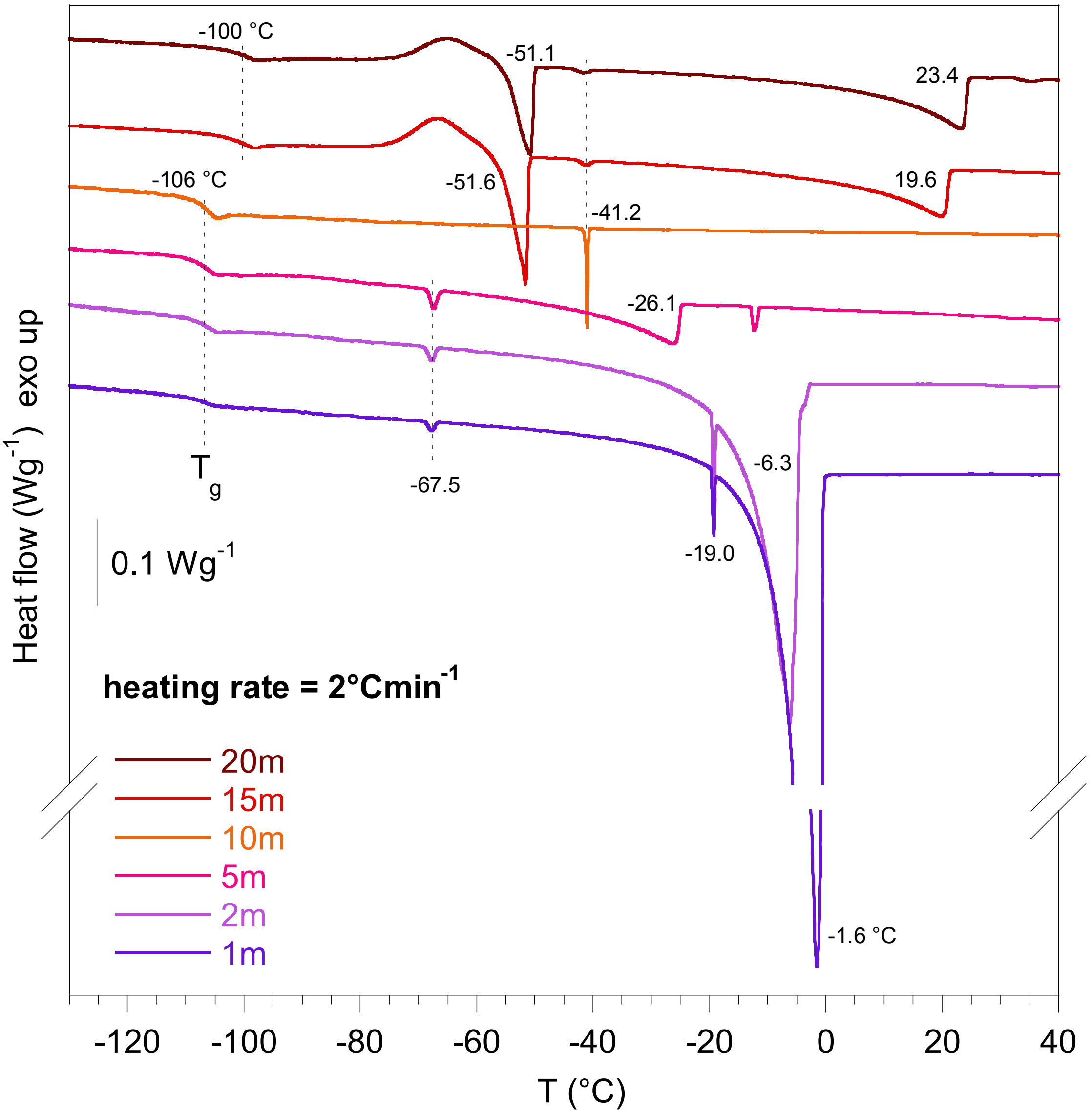

Figure S-1. DSC traces for LiIM14-H_2O system.

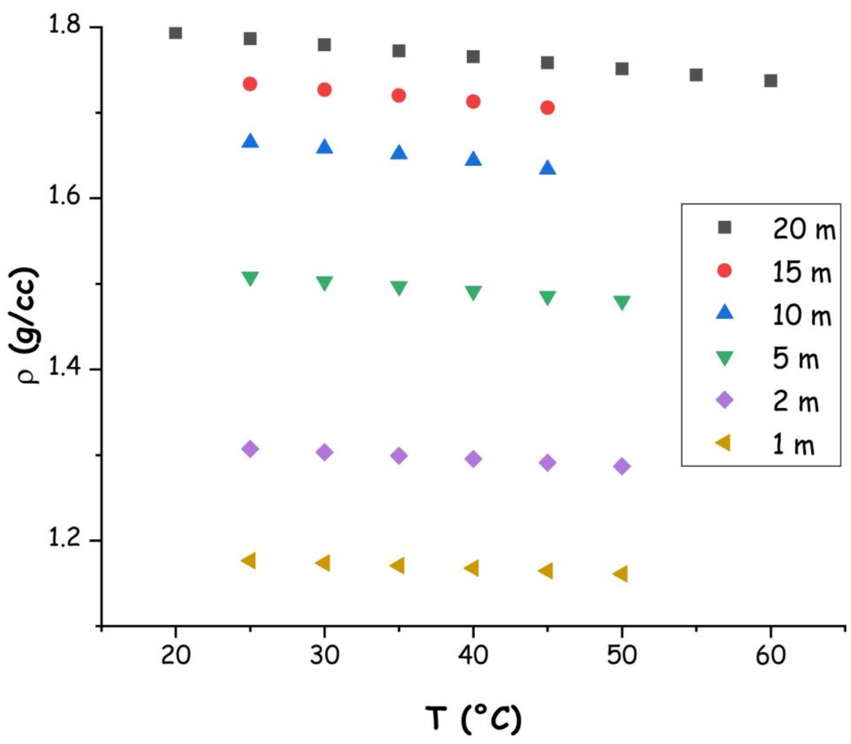

Figure S-2. Temperature dependence of density for the LiIM14-H_2O system, at different salt content.

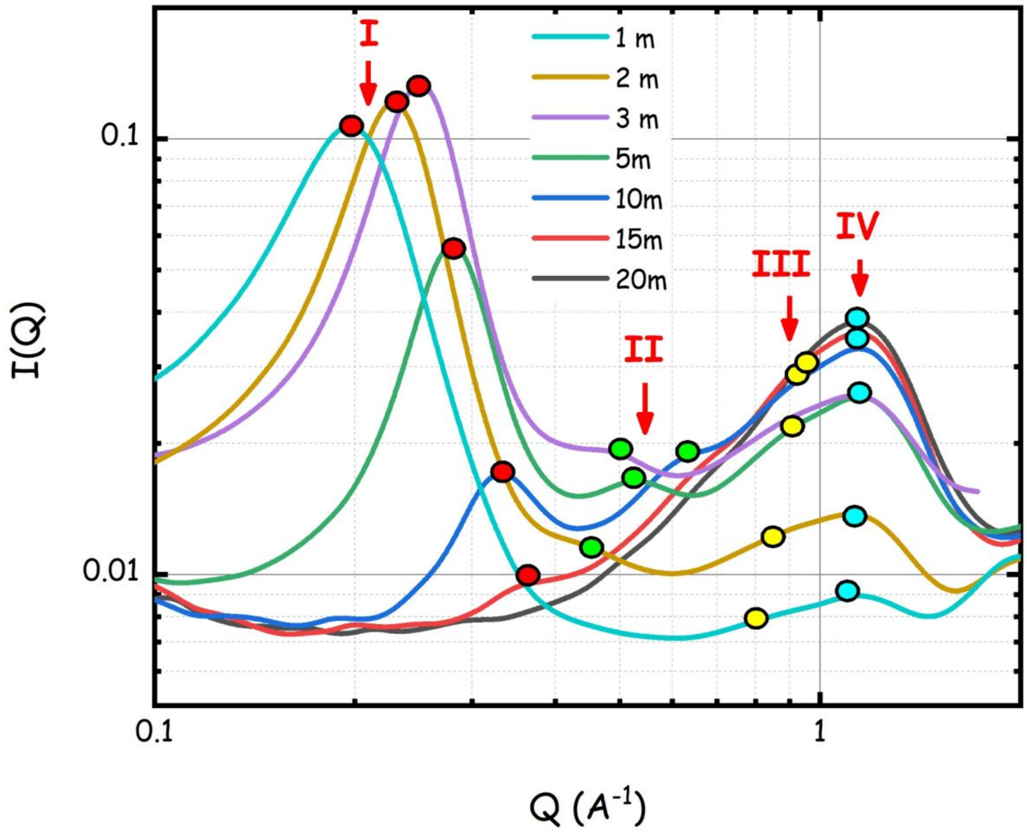

Figure S-3. Log-log version of the Small Angle X-ray Scattering patterns for the LiIM14-H_2O system at room conditions, as a function of salt content. The roman numbers refer to the four different peaks observed in the patterns. In the inset, the log-log salt concentration dependence for the characteristic size associated to peak I is reported. Differently coloured dots allow monitoring the concentration dependence of peaks positions

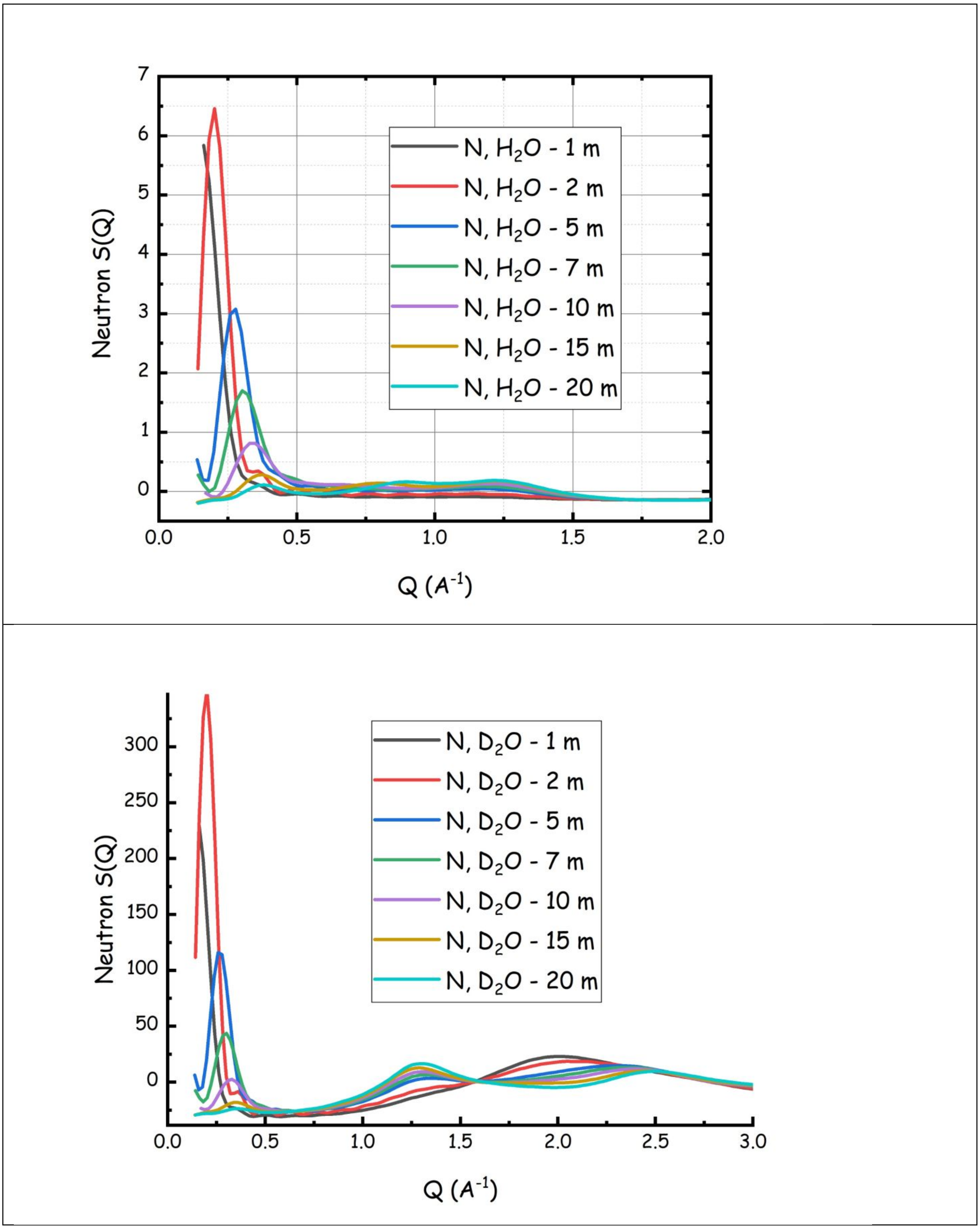

Figure S-4. MD-derived, neutron-weighted Wide Angle X-ray Scattering patterns from the (a) LiIM14-H_2O and (b) LiIM14-D_2O systems for different salt contents, at ambient conditions.

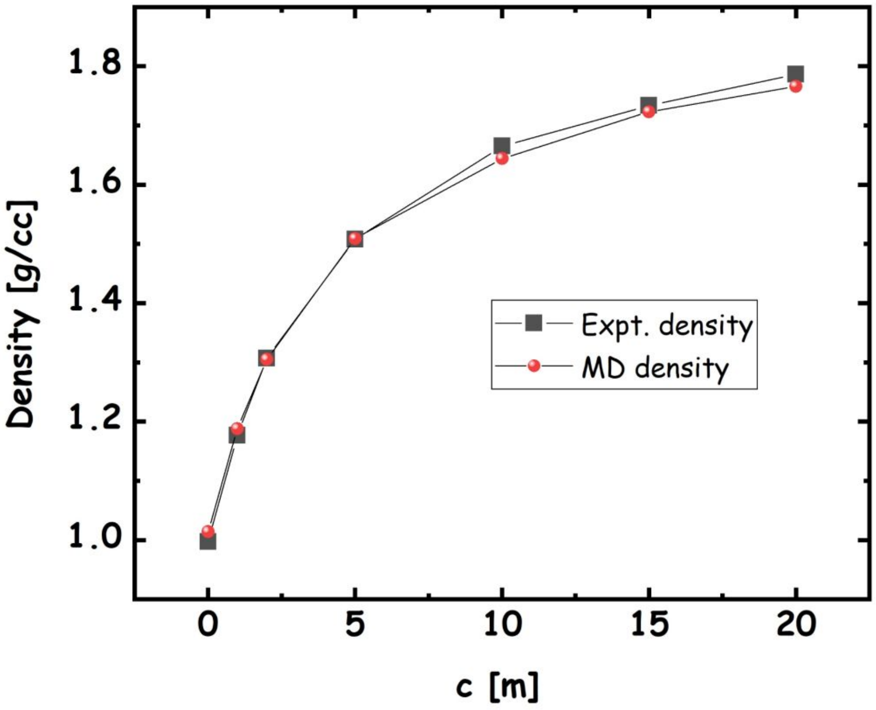

Figure S-5. Comparison between experimentally determined and MD-derived values for density of the LiIM14-H_2O system, at different salt content at 25°C.

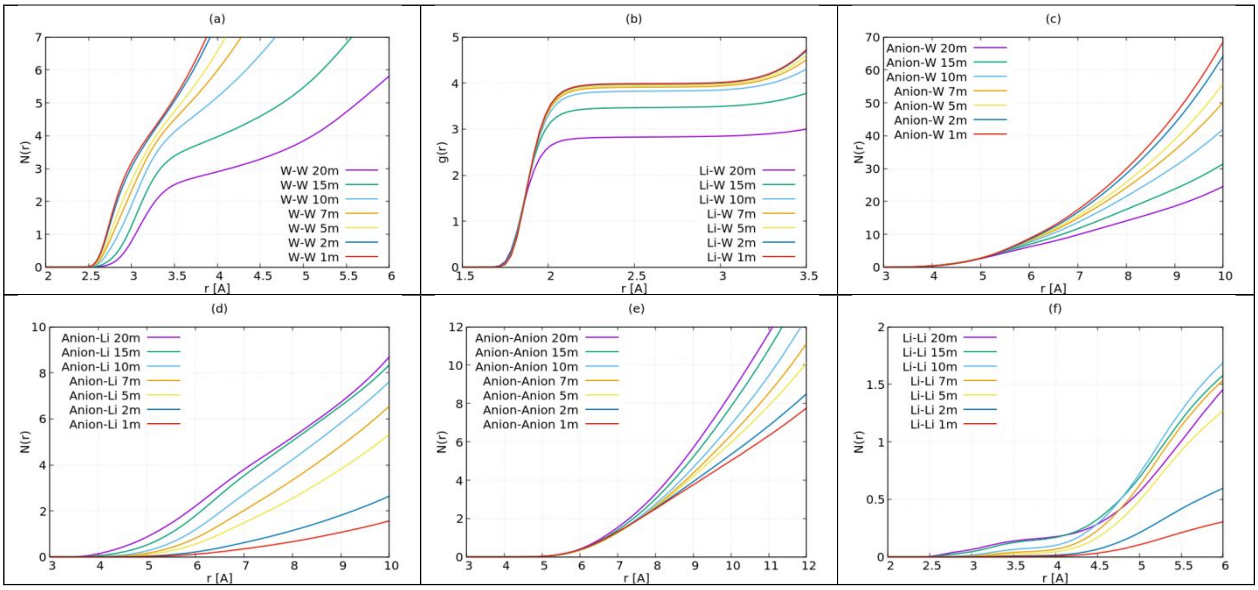

Figure S-6. MD-derived running coordination numbers derived from center of mass pair distribution functions for the different species in the LiIM14-H_2O systems, for different salt contents: a) water-water; b) lithium-water; c) anion-water; d) lithium-anion; e) anion-anion and f) lithium-lithium correlations are shown.

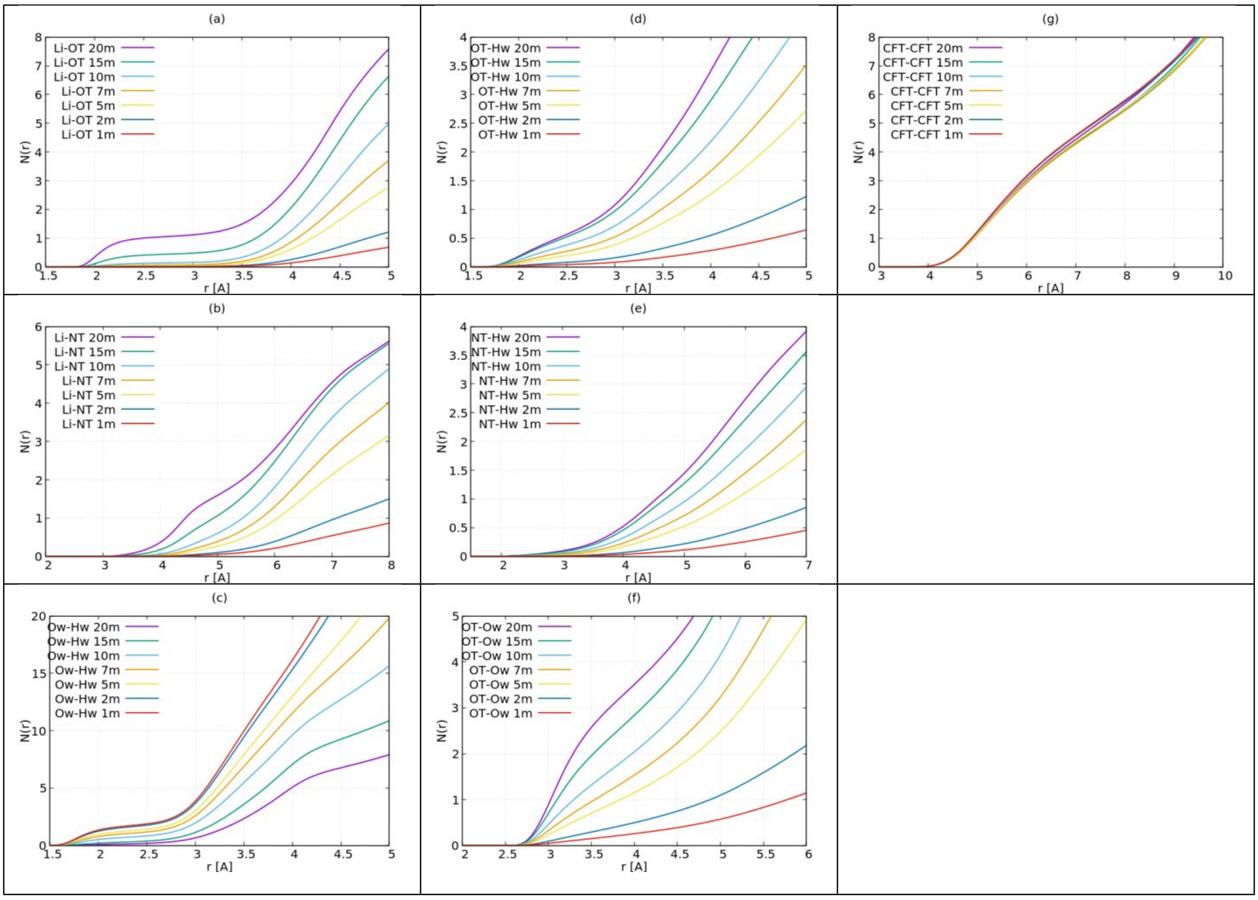

Figure S-7. MD-derived running coordination numbers of selected pair distribution functions for the different species in the LiIM14-H_2O systems, for different salt contents: a) Li-OT; b) Li-NT; c) Ow-Hw; d) OT-Hw; e) NT-Hw; f) OT-Ow and g) CFT-CFT correlations are shown. Ow and Hw refer to water's oxygen and hydrogen atoms.

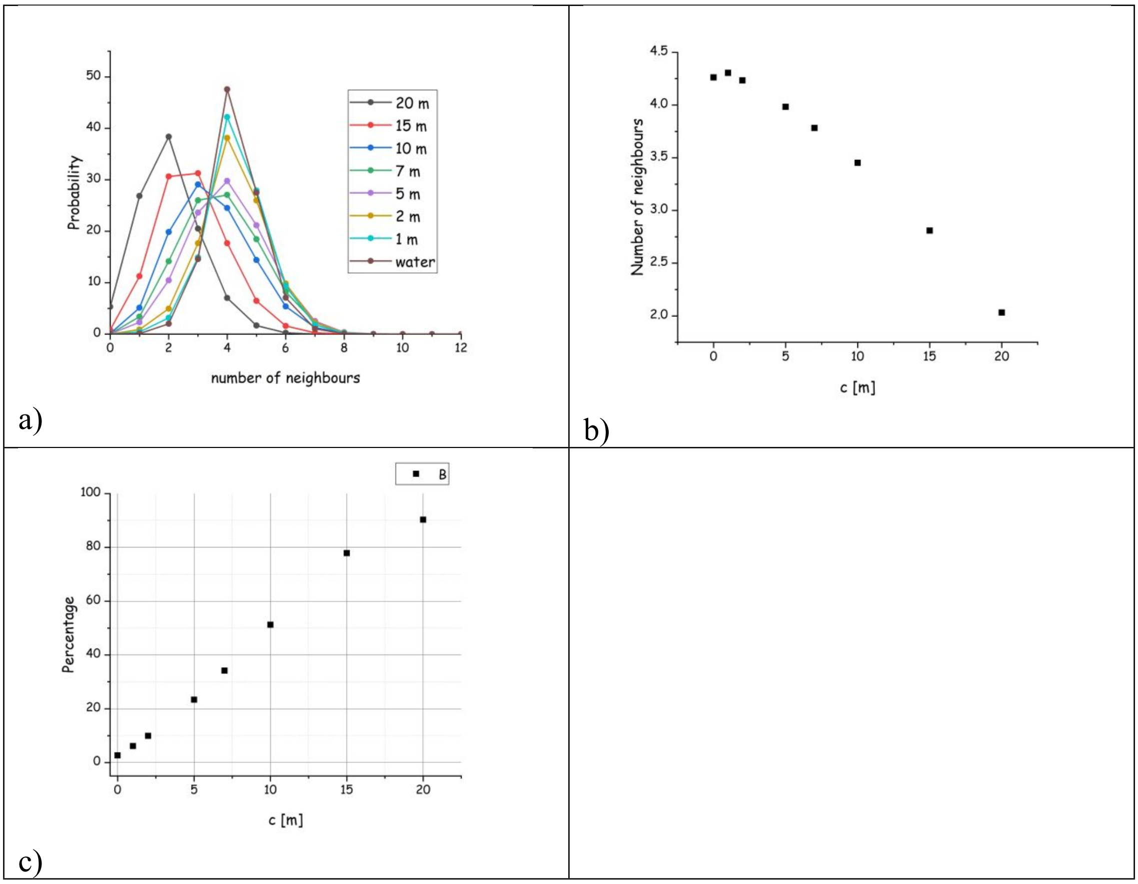

Figure S-8. Ow-Ow MD-derived solvation distributions: (a) Ow-Ow distribution of coordination number for Ow-Ow maximum distance equal to 3.3 Å; (b) average coordination number of water molecules surrounding a reference one; (c) Percentage of water molecules with no water molecules coordinated by hydrogen bonding.

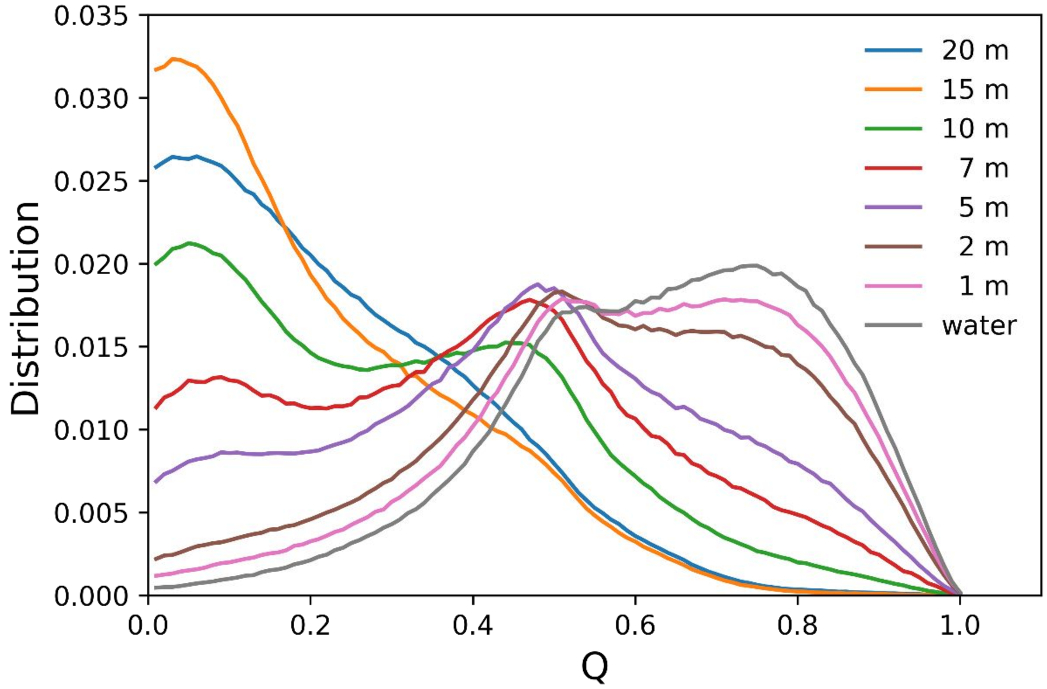

Figure S-9. Orientational tetrahedral order parameter, Q, obtained from MD simulations for the LiIM14-H_2O system, as a function of salt content.

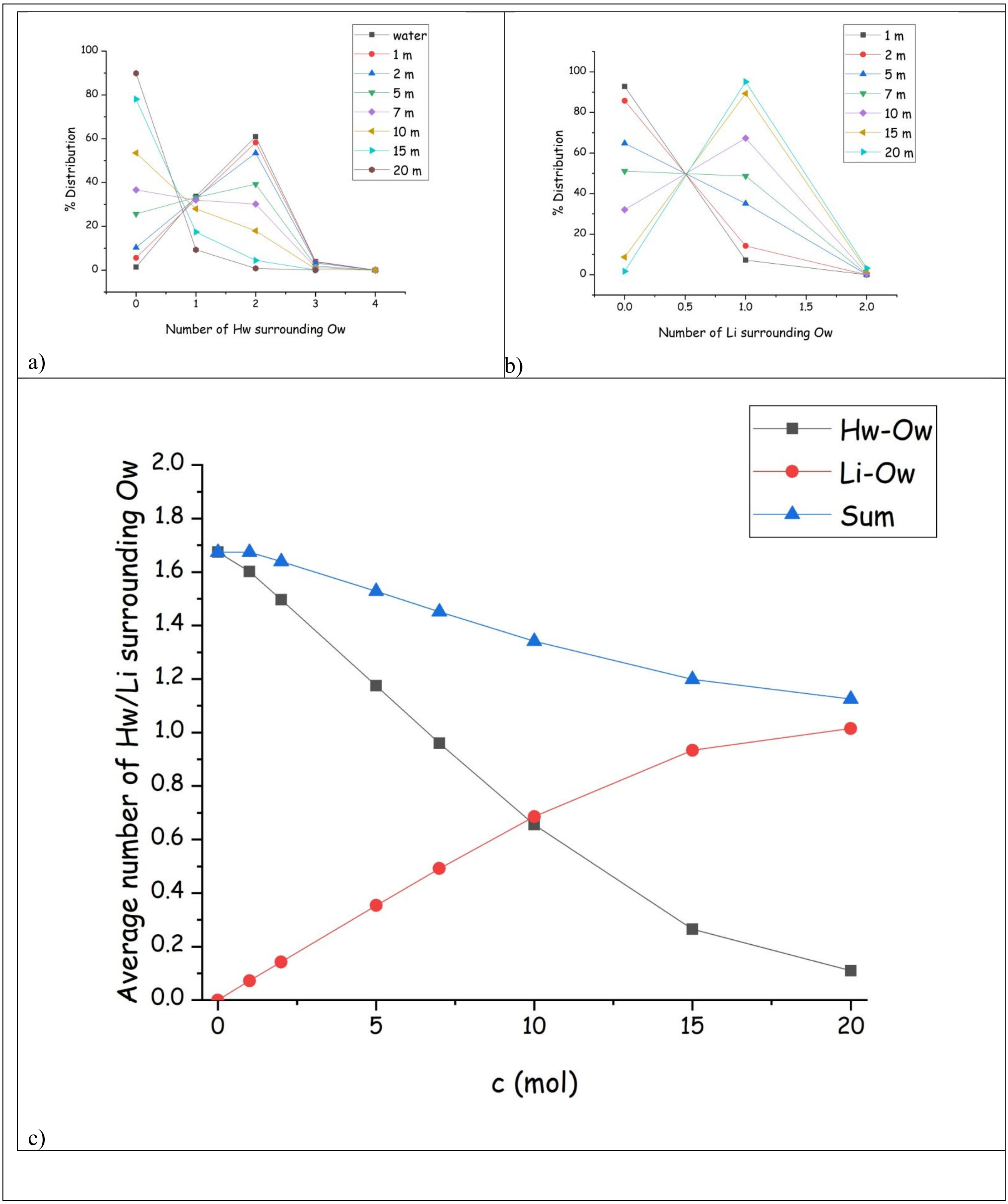
a)
% Distribution
Number of Hw surrounding Ow
water
1 m
2 m
5 m
7 m
10 m
15 m
20 m
b)
% Distribution
Number of Li surrounding Ow
1 m
2 m
5 m
7 m
10 m
15 m
20 m
c)
Average number of Hw/Li surrounding Ow
c (mol)
Hw-Ow
Li-Ow
Sum

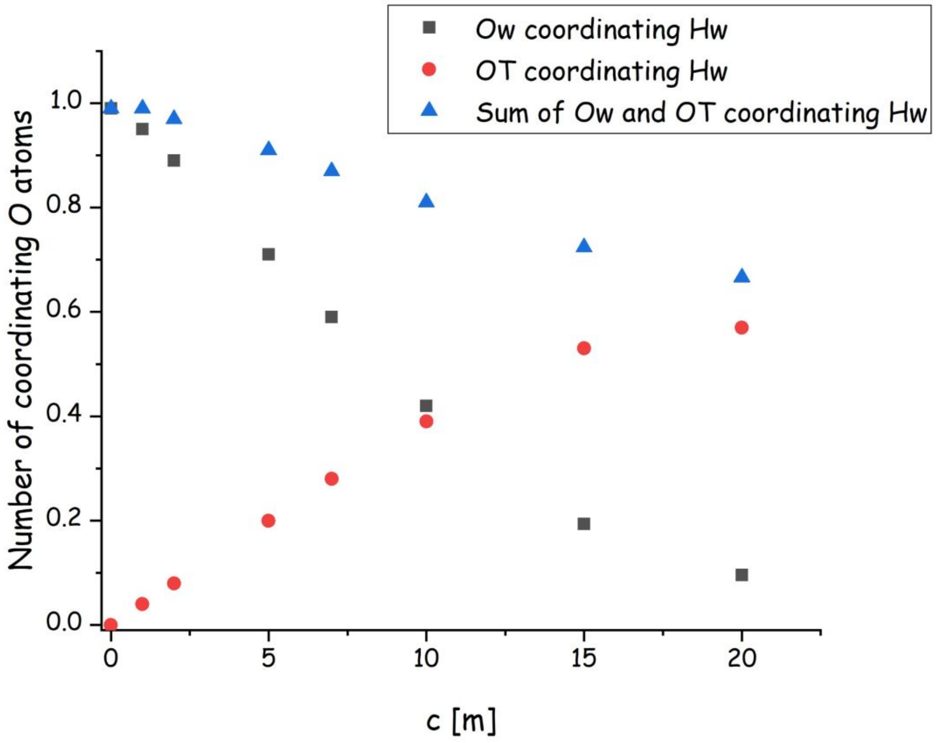

Figure S-11. Average coordination number of water (Ow) or anion (OT) oxygens surrounding a reference water hydrogen and their sum. From MD simulations of the LiIM14-H_2O system, as a function of salt content.

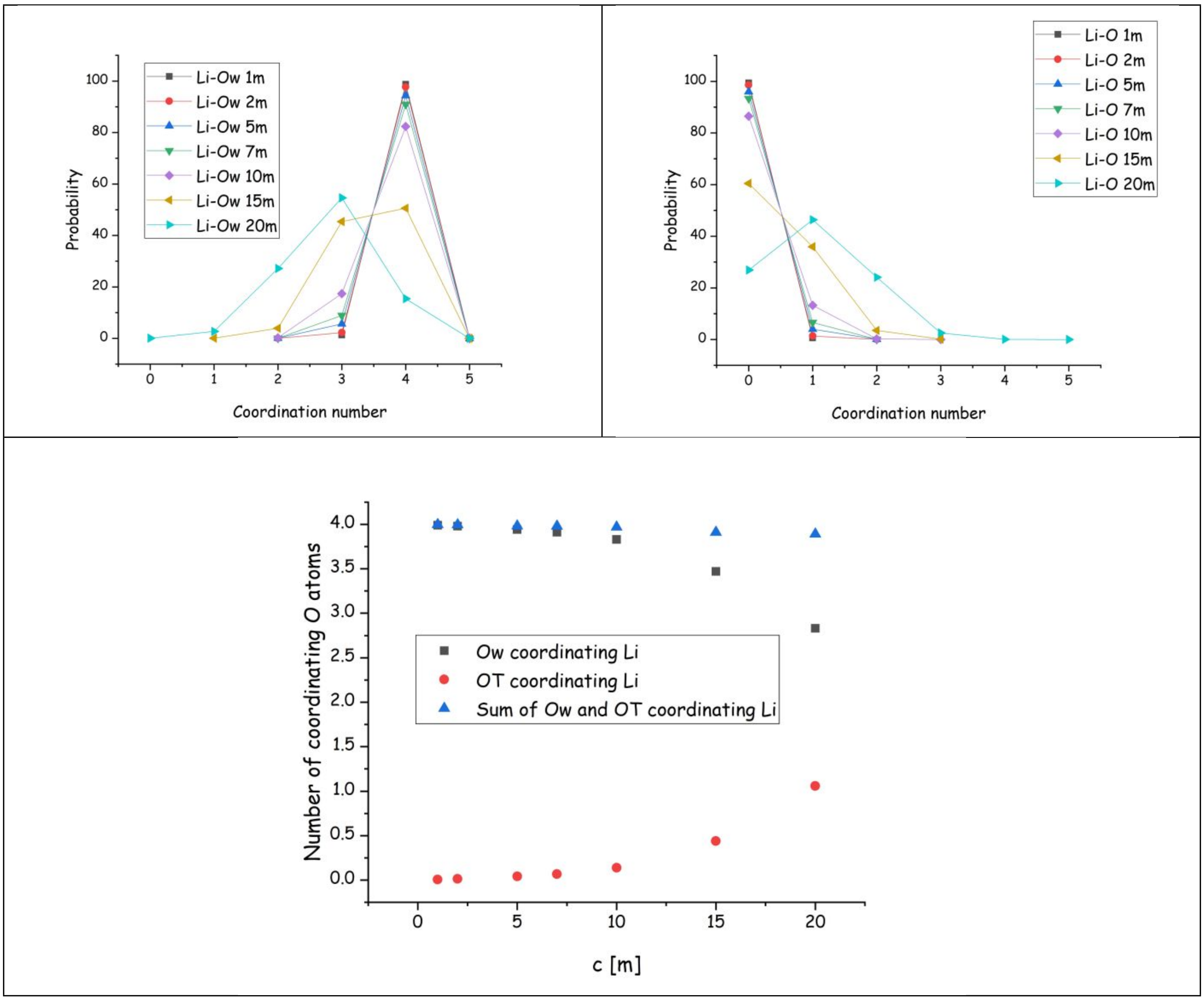

Figure S-12. (top, left) Li-Ow MD-derived solvation distributions; (top, right) Li-OT MD-derived solvation distributions; (bottom) average coordination number of water oxygens (Ow) or anion oxygen (OT) surrounding a reference lithium ion and their sum. From MD simulations of the LiIM14-H_2O system, as a function of salt content.

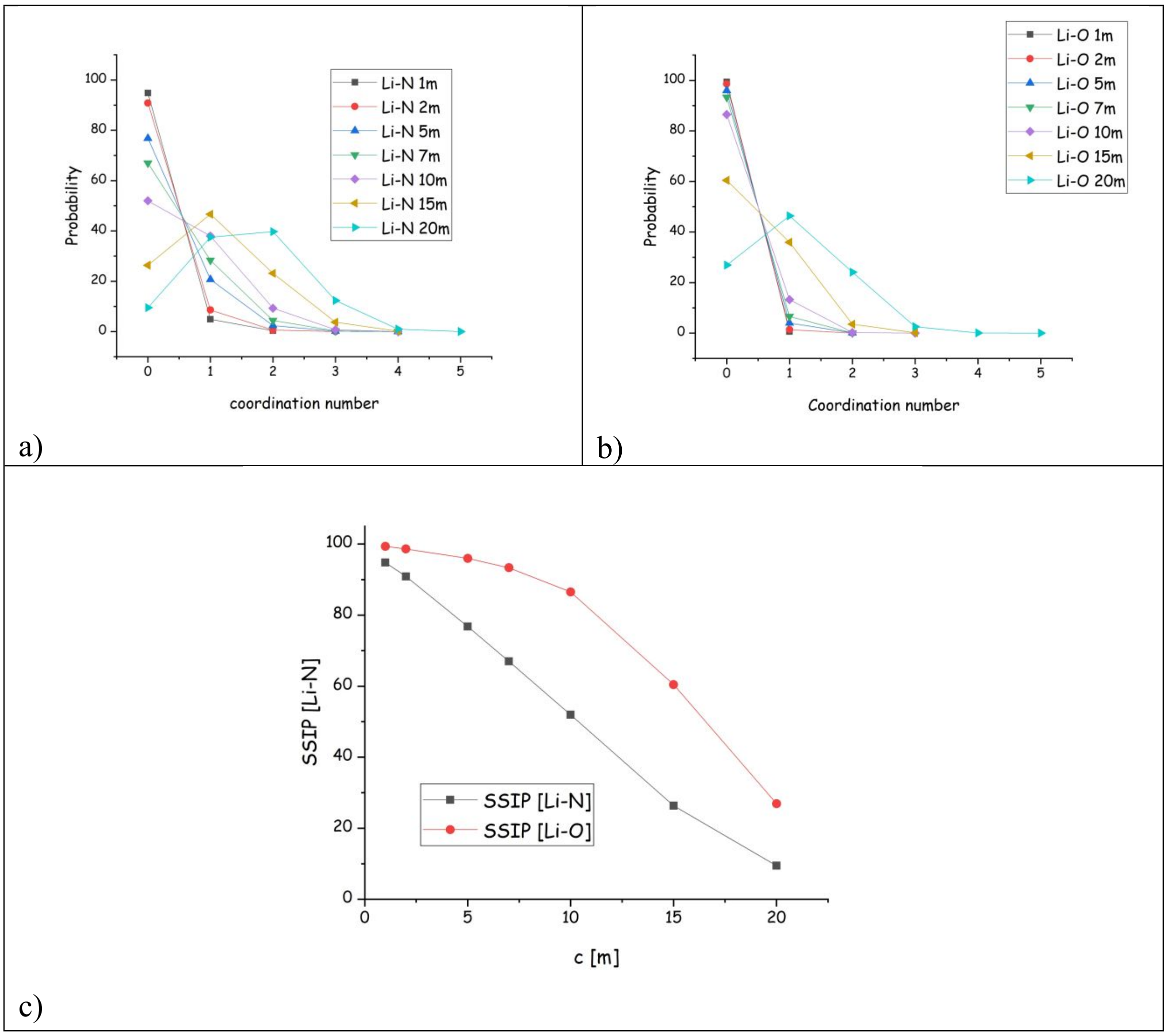

Figure S-13. (a) Li-N MD-derived solvation distributions; (b) Li-OT MD-derived solvation distributions; (c) Occurrence probability of solvent separated Ion Pair (SSIP), obtained as the probability of Li-N or Li-OT coordination number equal to zero from the above distributions. From MD simulations of the LiIM14-H_2O system, as a function of salt content.
